# 3D Computational Mechanics Elucidate the Evolutionary Implications of Orbit Position and Size Diversity of Early Amphibians

**DOI:** 10.1371/journal.pone.0131320

**Published:** 2015-06-24

**Authors:** Jordi Marcé-Nogué, Josep Fortuny, Soledad De Esteban-Trivigno, Montserrat Sánchez, Lluís Gil, Àngel Galobart

**Affiliations:** 1 Universitat Politècnica de Catalunya–BarcelonaTech, Terrassa, Spain; 2 Institut Català de Paleontologia Miquel Crusafont, Cerdanyola del Vallés, Spain; 3 Transmitting Science, Piera, Spain; University of Naples, ITALY

## Abstract

For the first time in vertebrate palaeontology, the potential of joining Finite Element Analysis (FEA) and Parametrical Analysis (PA) is used to shed new light on two different cranial parameters from the orbits to evaluate their biomechanical role and evolutionary patterns. The early tetrapod group of Stereospondyls, one of the largest groups of Temnospondyls is used as a case study because its orbits position and size vary hugely within the members of this group. An adult skull of *Edingerella madagascariensis* was analysed using two different cases of boundary and loading conditions in order to quantify stress and deformation response under a bilateral bite and during skull raising. Firstly, the variation of the original geometry of its orbits was introduced in the models producing new FEA results, allowing the exploration of the ecomorphology, feeding strategy and evolutionary patterns of these top predators. Secondly, the quantitative results were analysed in order to check if the orbit size and position were correlated with different stress patterns. These results revealed that in most of the cases the stress distribution is not affected by changes in the size and position of the orbit. This finding supports the high mechanical plasticity of this group during the Triassic period. The absence of mechanical constraints regarding the orbit probably promoted the ecomorphological diversity acknowledged for this group, as well as its ecological niche differentiation in the terrestrial Triassic ecosystems in clades as lydekkerinids, trematosaurs, capitosaurs or metoposaurs.

## Introduction

The usage of computational methods such as Finite Element Analysis (FEA) or Multibody Dynamics Analysis (MDA) to estimate the biomechanical performance of vertebrate skeletal and soft tissues has increased in the last ten years. Particularly, Finite Element Analysis [1] has been used in vertebrate palaeontology to simulate simplified 2D models or create high-resolution 3D models of vertebrates to study their function, morphological evolution, particular adaptation or constraints. (See [2] for a review of FEA methodology and examples). Other tools such as Parametrical Analysis (PA) are excellent inductive and non-invasive methods to test how morphological changes could affect the biomechanical performance of the biological structures, giving the keys to understand the evolutionary history and variability of the analysed organisms.

In vertebrate palaeontology, some previous works joined PA and FEA to test the behaviour and sensitivity of different parameters such as the material properties of the biological tissue [3], the homogeneity or heterogeneity of the bone [4], the sutures [5], or the influence of the loads applied [6,7]. Interestingly, FEA and PA were rarely used in vertebrate palaeontology to test how the variation of the original geometry affects the biomechanical performance [8].

**Fig 1:**
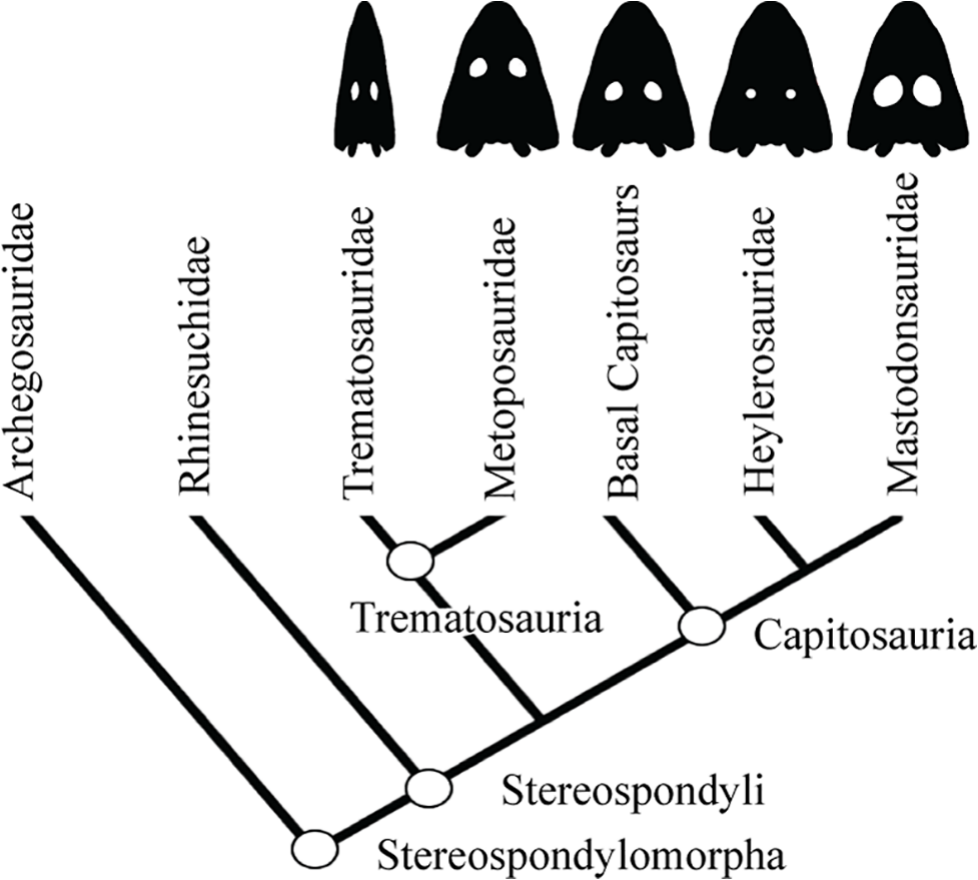
Simplified Cladogram of Stereospondyli based on Fortuny et al. [1] and Schoch [2].

Herein, we test the potential of joining Finite Element (FE) and Parametrical Analysis (PA) to test the effect of two different cranial parameters in Stereospondyls-one of the largest clades of the early tetrapods Temnospondyls- to evaluate its biomechanical role and evolutionary patterns. Members of Stereospondyls acquired medium to gigantic sizes being the top freshwater predators during the Permian and until the Middle Triassic. Its members were mostly characterized by dorsoventral flattened and strongly ossified skulls giving them a superficially crocodile-like appearance. Their shape diversity went from broad-headed (as *Alligator*) in the case of capitosaurs to slender headed (as gavialids) in the case of some trematosaurids. The ecomorphology of the Stereospondyls has been largely debated, specially focusing on their feeding ecology [9–14]. However, no previous studies have focused on the role of any morphological character that could be involved in the ecomorphology and feeding strategy of Stereospondyls, as have been largely performed in other groups such as crocodiles [15–18]. Thus, we analyse the role of the orbits in these top predators. Orbits position and size hugely vary among the different Stereospondyls groups (Fig 1); in metoposaurs, the orbits were positioned in a very anterior position in comparison with capitosaurs, while the orbit size also extremely varies among the Stereospondyls, with huge size in some capitosaur taxa (*Mastodonsaurus* sp. or *Eryosuchus* sp.) in comparison with other capitosauroid, trematosauroid or metoposauroid taxa (See [13,19] and references therein). The biomechanical role of these characters was analysed using Finite Element Analysis (FEA) and Parametric Analysis (PA) in a quantitative framework.

## Materials and Methods

### Sample

An adult skull of *Edingerella madagascariensis* was analysed. This taxon is well known from the Olenekian (Early Triassic) of Madagascar from several specimens, including ontogenetical series [20]. Phylogenetic analyses places this taxon as a basal member of the capitosaurian clade (See [20–22]. The specimen used as a case-study was described in detail by [22], being the largest and most well preserved specimen recovered to date for this taxon. The specimen comes from a siliceous nodule. A cast made with silicon resins allowed us to obtain a complete 3D skull with an exceptional fidelity of skull details, including most of the inner regions without deformation (See [22] for further details). The specimen analysed is stored at the Muséum National d’Histoire Naturelle (MNHN) in Paris, with the labelling MSNM V2992.

### Geometry

The skull of *E*. *madagascariensis* was digitalized using a medical CT scan Siemens Sensations-16, at 140 kV and 150 mAs giving an output of 512 x 512 pixels per slice. The pixel size and the inter-slice space were 0.586 mm and 0.1 mm, respectively. It was converted to a CAD model using reverse engineering techniques [23].

The digital model was treated with the software AVIZO, which enables to perform interactive visualization and computation on 3D data sets, and at the same time generates and modifies 3D surface models from stacked medical images such as CT, through image segmentation done in the STL (stereolithography) format involving the stages of segmentation and 3D reconstruction.

The reconstructed geometry had the usual irregularities in the surface due to the generation of the model from the CT scan. Consequently, the geometry required a transformation to achieve a geometry that fulfils requirements of quality, consistency and shape. The quality of tetrahedrals in the STL mesh is given by quality indicators of the mesh such as skewness and/or aspect ratio. The consistency refers to possible topological errors, and the shape to the final geometry obtained with smooth operations. This was done using the automatic thresholding tools of the CT-software AVIZO in a first step, and in a second step, in those areas where the irregularities were present, a semi-automatic thresholding approach to correct them with smoothing and relaxation tools, to finally generate the 3D model. A refinement and smoothing and/or a relaxation of some regions of the geometrical mesh was also required. It is important to differentiate between the geometrical and the Finite Element (FE) meshes: these meshes were generated by different algorithms and with different functions. The geometrical mesh was only used to obtain the digital model from the CT scan, while the FE mesh is used to solve the equations of the structural analysis problem.

**Fig 2:**
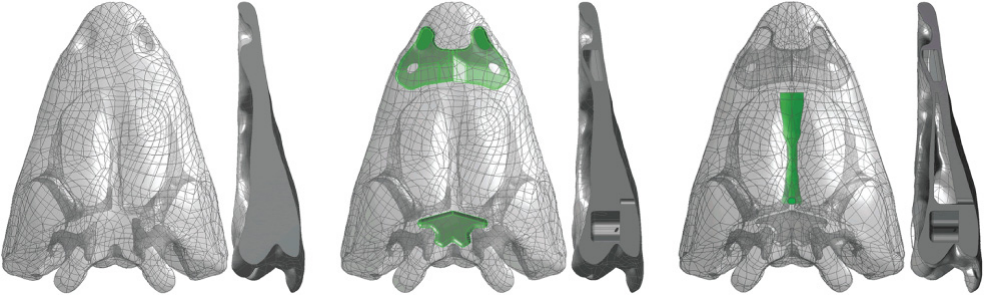
Skull of *the Edingerella madagascariensis* analysed showing the inner regions and cavities.

After this part in AVIZO, some inner regions were reconstructed (i.e. the space between the vomer and the skull roof bones, the endocranial area and the inner part of the cultriform process). These regions are rarely preserved in 3D in the fossil record (e.g. vomer and skull roof bones use to be in contact due to taphonomical factors). Despite this, reconstruction can be done because in the literature these inner regions are described [24–26], but also thanks to personal observations of one of us (J.F.). These features were reconstructed using the CAD interface of the Finite Element Package ANSYS 14.5 (Fig 2).

Additionally, in order to evaluate the overall skull shape diversity within Stereospondyls the original model was wrapped in two different directions generating a long-slender snouted morphotype and a broad skull and short-snouted morphotype. This was done to evaluate how the shape morphology affected the Von Mises stress patterns (See S2 Document) and revealed that the general patterns found in the original model were also present in the additional geometries generated (See S3, S4, S5 and S6 Figs).

### Finite Element Analysis

**Fig 3:**
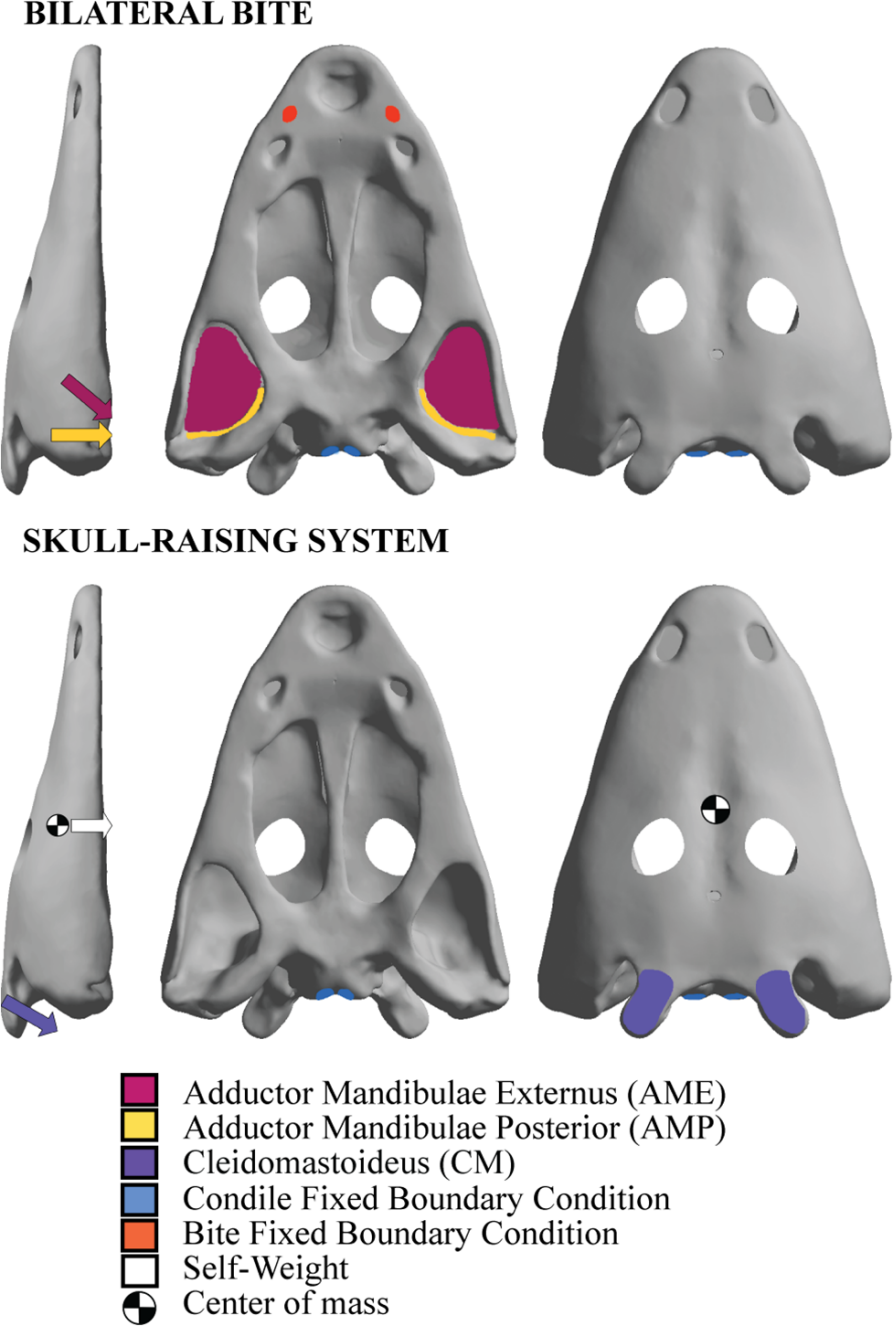
Loadings and boundary conditions on the skull model for the a) bilateral bite and b) skull-raising system.

A Structural Static Analysis was performed using the Finite Element Package ANSYS 14.5 in a Dell Precision Workstation T7600 with 32 GB of RAM (4X8GB) and 1600 MHz. Two different cases of boundary and loading conditions were evaluated in order to quantify stress response and deformation. The first one represented a bilateral bite and the second one the raising skull system (Fig 3, based on [10]). Therefore, the first loading case simulates the direct bite on prey, as known for some extant salamanders, as these animals probably used bilateral loading instead of a unilateral biting (as is also known for extant crocodiles). As previously reported [9], the skull raising system simulated the stress distribution during the opening of the mouth.

For the bilateral loading, the Adductor Mandibulae Externus (AME) and the Adductor Mandibulae Posterior (AMP) were considered in the model according to this soft tissue reconstruction based on several authors (e.g. [27,28]). Regarding the Adductor Mandibulae Internus (AMI), an additional analysis was performed (See S1 Document), revealing that this muscle has null influence on the bilateral loading stress and displacements. Consequently it was not included in the analysis. The muscular insertion areas of AME and AMP were defined in the model in order to apply the forces of the muscular contraction during the prehension/bite. The direction of these forces was defined by the line that joins the centroid of the insertion area in the skull with its correspondence in the insertion area of the lower jaw. According to [29] 0.3 MPa were assumed as muscular contraction pressure (Force per unit area) applied at the insertion area of each muscle.

Fixed boundary conditions were applied at the occipital condyles (as related to vertebral column in the living animal) and the bite point was positioned close to the premaxilla/maxilla suture, where fangs were actually present.

The skull-raising system case simulates a bilateral skull-raising system at the tabular bones-which has a characteristic horn morphology- in the y-direction to induce the elevation of the skull. Considering the cortical bone density to be 2 g/cm^3^, the self-weight of the skull was included with the volume [10]. The opening of the mouth was simulated adding a force that opens the mouth balancing in the opposite direction the self-weight of the skull to a null displacement

A fixed boundary condition was applied in the tabular horn considering, on one hand, the triple suture between the tabular, the post-parietal, and the supratemporal, and on the other hand, the posterior edge of the tabular. Fixed boundary conditions were also applied at the occipital condiles.

Although bone properties for extinct vertebrate models have been discussed since the first FEA analyses (see [2] for a review), according to the sensitivity analysis of [7] results for heterogeneous materials closely match the results for homogeneous materials and are appropriate for a comparative analysis [30]. Consequently, elastic, linear and homogeneous material properties were assumed for the bone of *Edingerella madagascariensis*, using the following values: E (Young’s modulus): 6.65 GPa and m (Poisson’s ratio) 0.35. These values for *Crocodylus* frontal and prefrontal bones are based on the work of [31].

The usage of these arbitrary values did not lead to realistic results, but provided a good estimation of the stress distribution on the skull because this distribution will not vary neither under Young’s modulus nor under Poisson’s ratio values [1]. Regardless of this, the use of the same linear and homogeneous material properties in a comparative analysis for different specimens has been demonstrated useful for studying biological implications [8]. The skull was meshed with an adaptive mesh of hexahedral elements [32] around 450000–500000 nodes depending on each parametric case.

### Parametric Analysis

The 3D analyses performed in the skull of *Edingerella madagascariensis* mixed PA and FEA. The boundary conditions applied to the different biting cases included bilateral bite and the skull raising system (see below). The PA was done by modifying the values of two variables separately: the position of the orbits along the principal axis of the skull and the size of the orbits. The influence of changes in the orbit in the values of maximum Von Mises stress and displacements, as well as stress values at certain points, was observed and analysed. As revealed by the fossil record, the variability of orbit size and position in this group was very high, in contrast with other characters (as position of the Pineal foramen) that were almost invariable along the evolutionary history of Stereospondyls [19]. On this regard, the orbit position could be analysed according to the distance between the orbits and the pineal foramen and in relation to the total length of the skull (Table 1, Fig 1). Some taxa presented these two structures very close to each other (e.g. 0.09, *Eocyclotosaurus* and related forms, [33] in clear contrast with other taxa with widely separated orbits and pineal foramen (e.g., more than 4 times) as found in *Metoposaurus* and related forms [34].

**Table 1:** Distances relating skull length, pineal foramen position and orbit diameters in different Stereospondyls.

Taxon	Skull length [mm]	distance pineal foramen–orbit [mm]	distance pineal foramen orbit / skull length	maximum orbit diameter [mm]	maximum orbit diameter / skull length
*Metoposaurus*	314	131	0.4171	40	0.1273
*Benthosuchus*	160	25	0.1562	20	0.125
*Cyclotosaurus*	255	30	0.1176	27	0.1058
*Eocyclotosaurus*	305	27	0.0885	25	0.0819
*Wantzosaurus*	325	64	0.1969	47	0.1446
*Edingerella*	130	18	0.1384	22	0.1692
*Mastodonsaurus*	520	116	0.2230	1190	0.2288

Data extracted from published specimens for each taxon: *Metoposaurus* [48]*; Benthosuchus*, *Cyclotosaurus and Eocyclotosaurus* [33]*; Wantzosaurus* [35]*; Edingerella* [22]*; Mastodonsaurus* [36].

**Fig 4:**
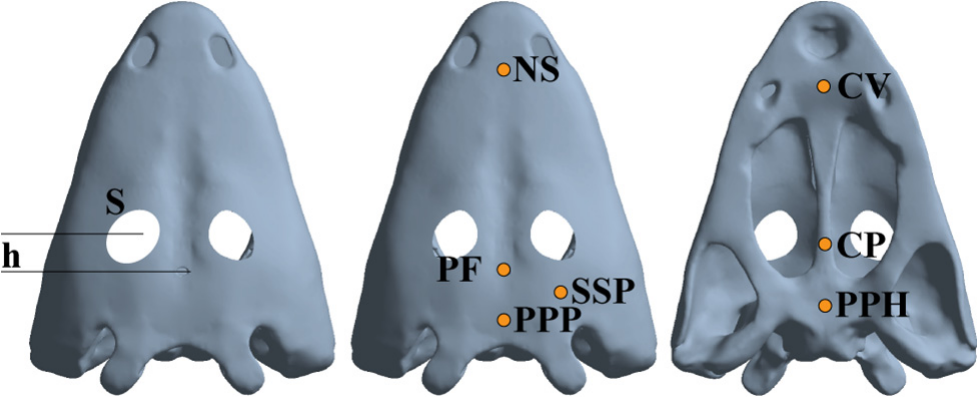
Variables S and h used in the parameterization. Dorsal and ventral views of the skull model of *Edingerella madagascariensis* with the points where the Von Mises Stress was recorded for a bilateral bite and a skull-raising system analysis. See text for abbreviations.

Similarly, the size of the orbits also presents high variability. Considering the maximum diameter of the orbit in relation to the total length of the skull, some taxa present relatively small sized orbits (e.g. 0.08, *Eocyclotosaurus* and related forms [33], being different from most taxa with larger orbits (e.g. 0.12, *Benthosuchus* or *Wantzosaurus*, [33,35] or those with extremely big orbits (e.g. 0.23, *Mastodonsaurus* or *Eryosuchus*, [19,36] being almost 3 times the size of the smallest ones. Therefore, the parameterization analysis simulated the variability of these parametres (Fig 4). Value h corresponded to the distance between the centre of the pineal foramen and the centre of the orbit. The original distance from the centre of the pineal foramen to the centre of the orbits was h = 17.5 mm. The distances ranged from from 2.5 mm to 42.5 mm in steps of 2.5 mm for each case solving 18 cases in total. Value S corresponded to the proportion of the orbit size, considering 1 the original size. The size proportion of the orbits that was analysed went from 0.125 to 1.625 in steps of 0.125, solving 14 cases in total. These cases overcome the variability range found in the fossil record to test if the results varied and explore any biological/biomechanical explanation for cases not found in fossil record.

Von Mises stress distribution was recorded because, according to [37], the Von Mises criterion is the most used in the cortical bone when isotropic material properties are assumed. Stress data were recorded for different parts of the skull (Fig 4). These were: a) the joint point between the parietals and postparietals (PPP), b) the Pineal Foramen (PF), c) the cheek area, in the triple suture joint of the squamosal, supratemporal and postorbital (SSP), d) in the middle area along the nasals suture (NS), e) the middle point of the Parasphenoid (PPH), f) the posterior area of the Cultriform process (CP) and g) the central area of the vomer (CV).

### Correlation of the parameters

In order to deeply understand the behaviour of the PA in terms of the results obtained, a correlation between the stress values at the recorded points and the size of the orbit (S) or distance (h) was developed.

We adjusted an Ordinary Least Squares (OLS) regression, because in this model the response variables are random, whereas the predictor variable represents fixed values chosen by the researchers [38], as in the present case. Therefore, S and h were considered as the predictor variable and the stress values at each recorded point were the response variables. Homoscedasticity of residuals was checked using the Breusch-Pagan statistic, a test for heteroscedasticity, i.e. nonstationary variance of residuals [39]. Heteroscedasticity is present when the size of the error term differs across values of an independent variable. Lack of homoscedasticity strongly violates the assumptions of the regression model, so the statistics derived from it are not reliable. To measure the proportion of the total variation in the stress values that is explained by its linear relationship with the predictor variable (S or h), the coefficient of determination (r2) was used. These analyses were developed using PAST v. 3.04.

Other correlation coefficients have been developed to be more robust than the Pearson correlation and more responsive to nonlinear relationships [40]. Spearman's rank correlation coefficient is a nonparametric measure of mathematical dependence between two variables. Therefore, Spearman's rank correlation coefficient was also used to assess the correlation between those variables where a non-linear relationship was suspected.

### Multivariate Analysis

The skull is a single structure and it was interesting to analyse the response of its stress levels at the different points in a multivariate manner. To achieve this goal, we developed Principal Components Analyses (PCAs) for each of the analysed cases.

The Principal Components Analysis enables the reduction of the dimensionality of the data, as well as revealing patterns that cannot be found by analysing each variable separately. It transforms the original variables into a new set of uncorrelated variables called Principal Components (PC). The simplest way to understand PCA is in terms of axis rotation. PCA can be viewed as a rotation of the principal axis, so that the new axis explains as much of the variance as possible, with the second PC being orthogonal to it (Quinn and Keough, 2002). PCA can be developed using covariance or correlation matrix. The covariance matrix is based on mean-centered variables and is appropriate when the variables are measured in comparable units and differences in variance between variables make an important contribution to interpretation, as in this case.

## Results

**Fig 5:**
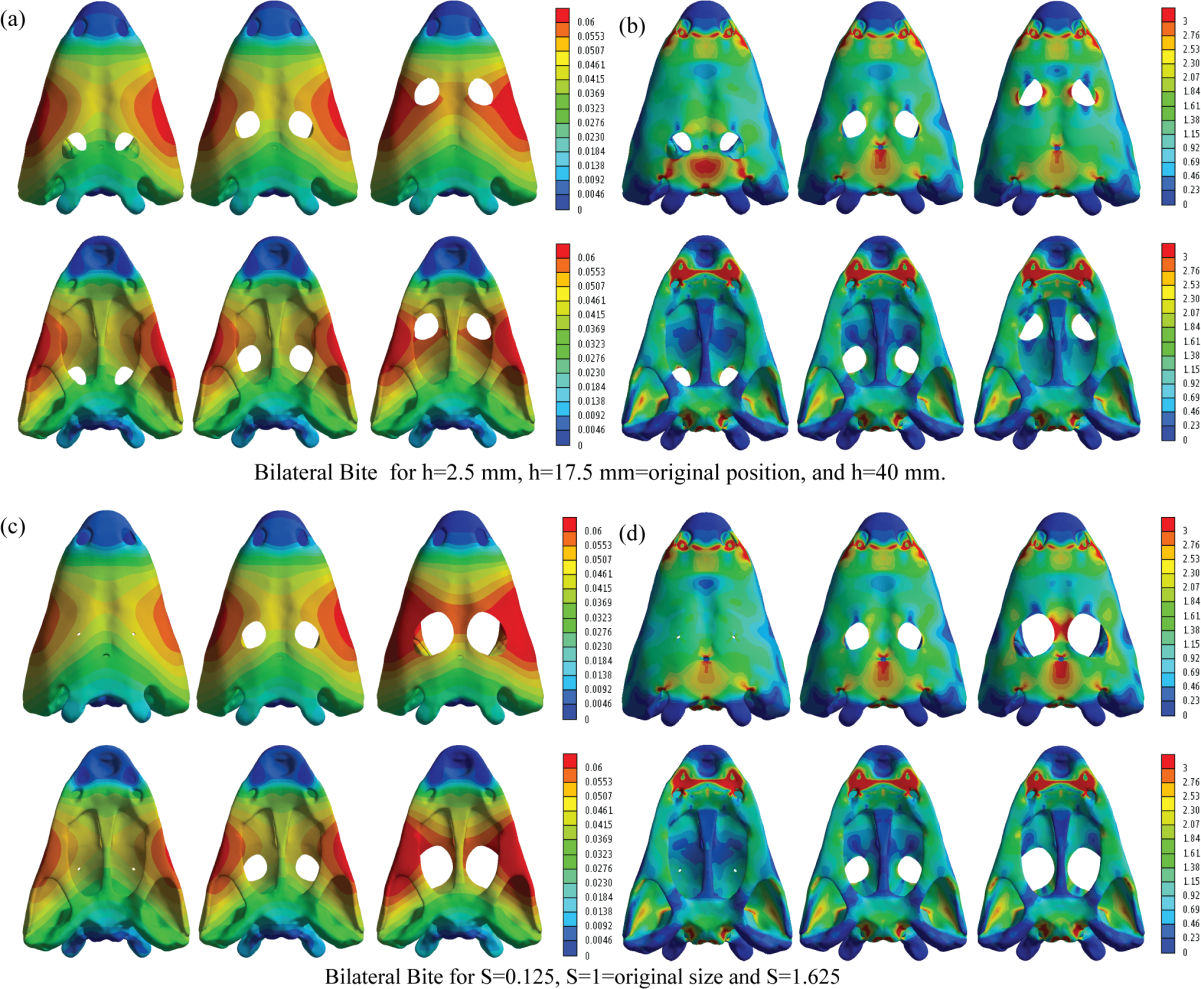
Displacements (mm) and equivalent Von Mises stresses (MPa) in bilateral bite for (a) (b) h = 2.5 mm, h = 17.5 mm and h = 40 mm and (c) (d) S = 0.125, S = 1 and S = 1.625.

**Fig 6:**
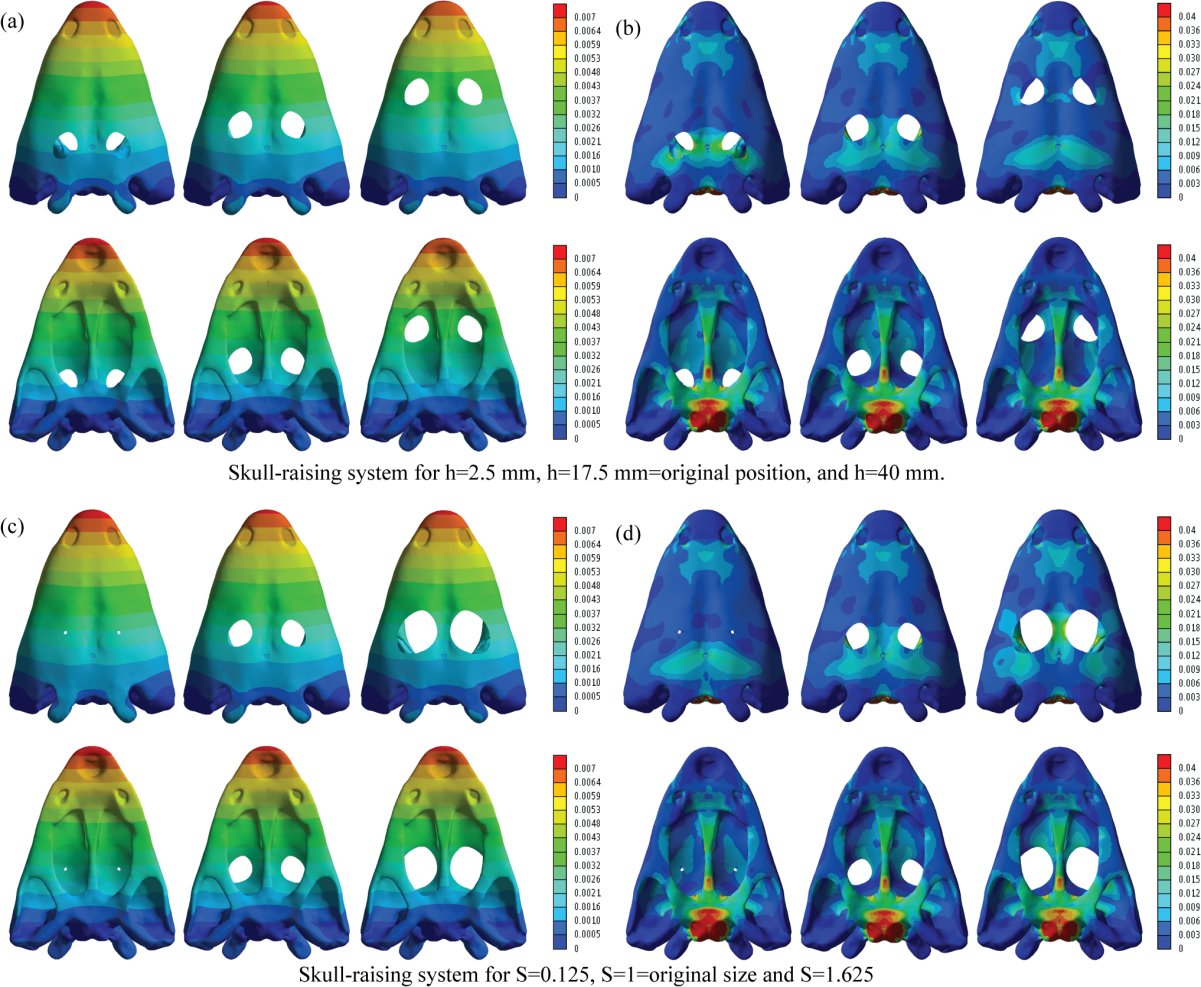
Displacements (mm) and equivalent Von Mises stresses (MPa) under a skull raising loading for (a) (b) h = 2.5 mm, h = 17.5 mm and h = 40 mm and (c) (d) S = 0.125, S = 1 and S = 1.625.

The stress values of the skull for the bilateral bite and the skull-raising system were obtained from FEA. The coloured maps of the displacements and Von Mises Stress are shown for the skull model (Figs 5 and 6). Specific values were obtained on the points previously described (Fig 4).

**Fig 7:**
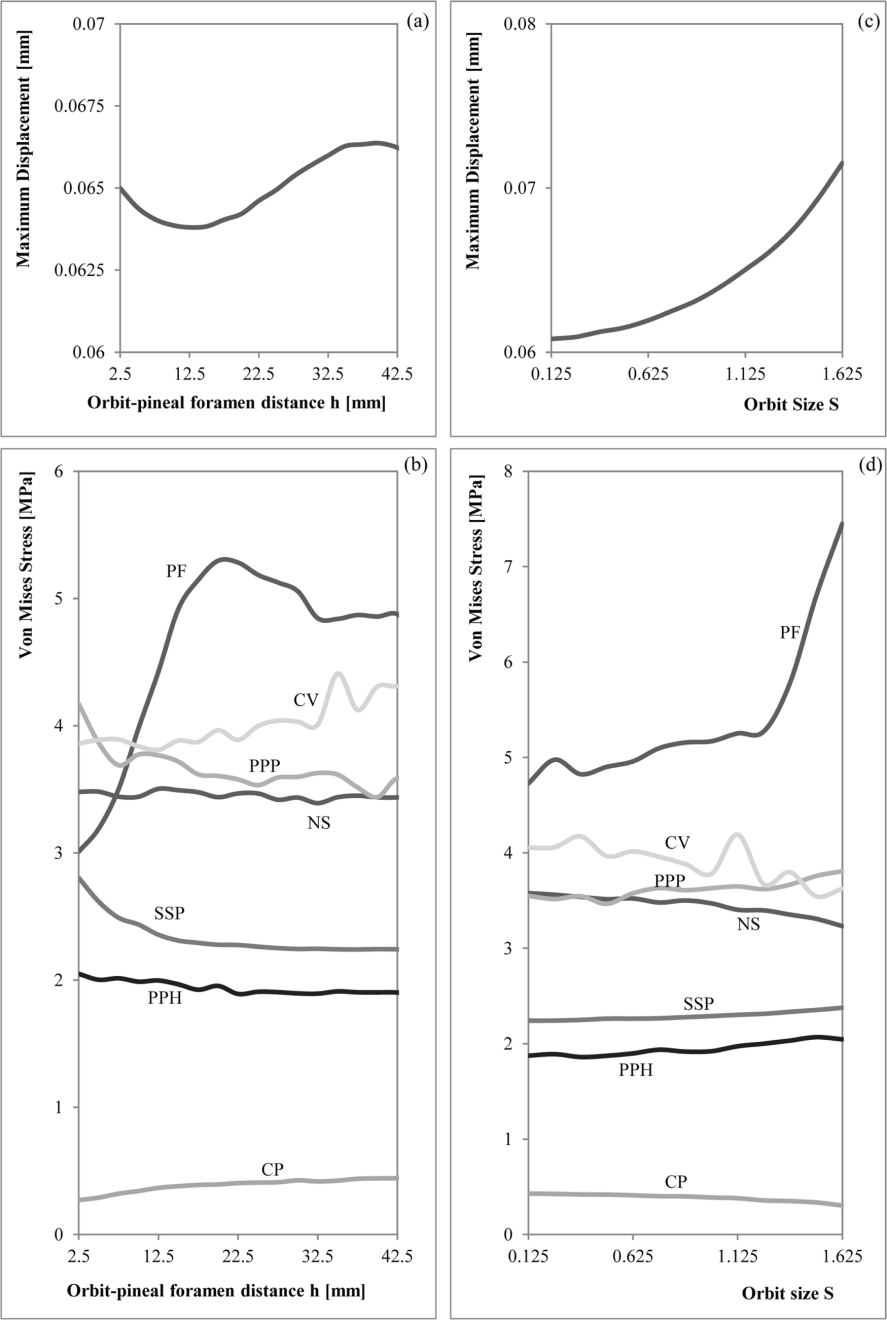
Graphical relationship between the Orbit-pineal foramen distance with the (a) maximum displacement and the (b) Von Mises Stresses and the Orbit Size with the (c) maximum displacement and the (d) Von Mises Stresses under a bilateral bite.

**Fig 8:**
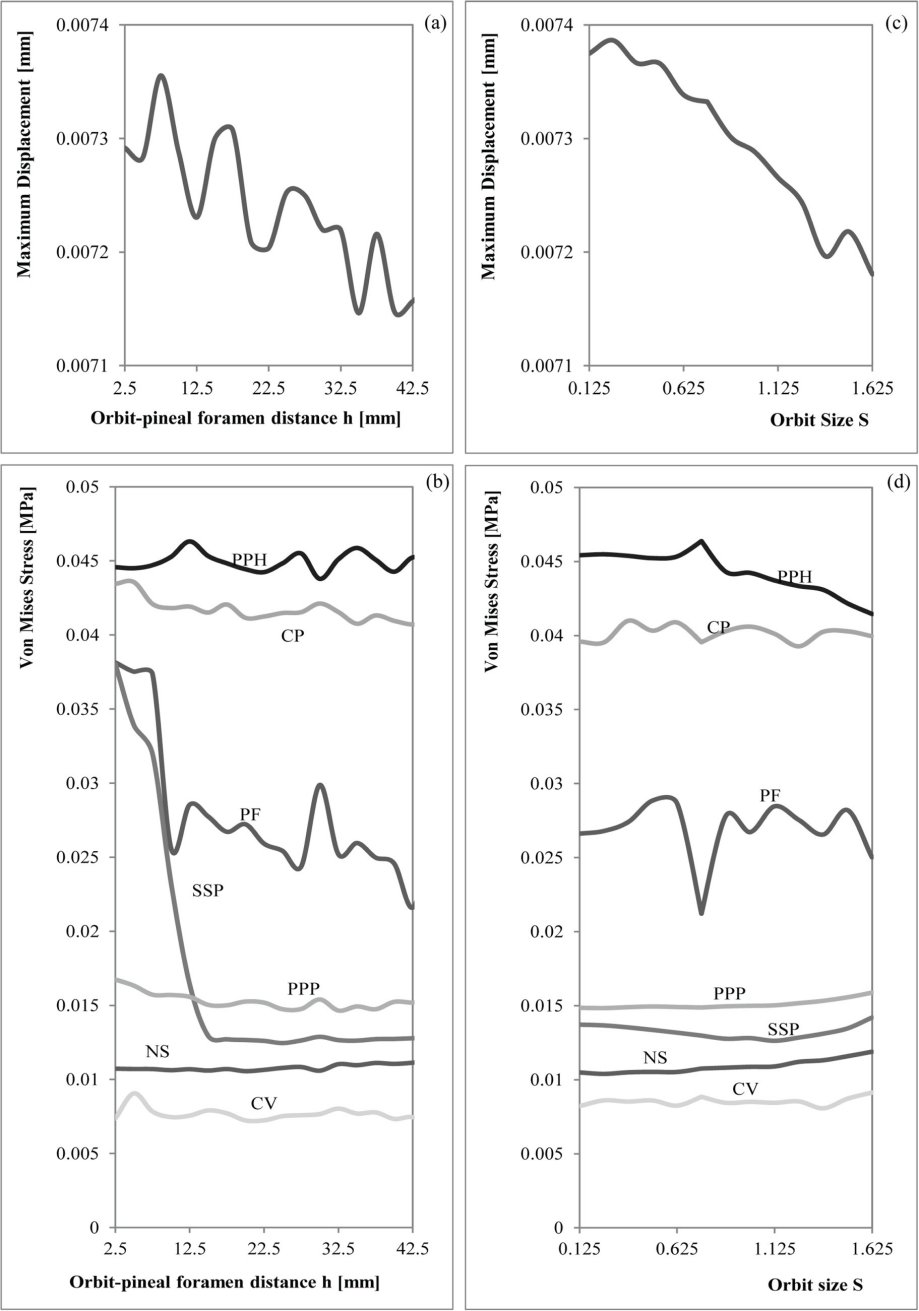
Graphical relationship between the Orbit-pineal foramen distance with the (a) maximum displacement and the (b) Von Mises Stresses and the Orbit Size with the (c) maximum displacement and the (d) Von Mises Stresses during the skull raising.

For the bilateral loading, S1 and S2 Tables show the numerical results of Von Mises stress and displacements obtained for the parameterization of the position of the orbits (h) and the size of the orbit (S), whereas S3 and S4 Tables show the percent differences of the values of the modified geometry respect the original geometry (S = 1 and h = 17.5 mm). S5, S6, S7 and S8 Tables show the same results for the skull-raising system respectively. Figs 7 and 8 show the graphical representation of these values.

### Bilateral Bite

#### FEA and PA

The maximum value of displacement is almost uniform and slightly varies when the position (h) and the size proportion (S) of the orbit are changed (in most of the cases less than 5% (See S3 and S4 Tables)). There is a small increment of the differences (7% and 10%) on the values of displacement when the size (S) of the orbit is extremally big. Modifying the position (h) and size (S) of the orbits does not affect the distribution of the displacements on the skull (Fig 5) and the location of the maximum value. Regarding the displacements, the highest values were located in the cheek region of the skull.

The Von Mises stress peaked around the otic notch in all the cases (Fig 5), thus not depending on the size and position of the orbits. In the parameterization of the orbit position, stress differences are only present on the skull roof, showing that stress decreases on the posterior part of the skull and increases on the interorbital area when the orbits are placed on an anterior position. In the case of the size proportion of the orbit, stress differences between the different cases show that the magnification of orbit size causes higher stress levels in the interorbital region. However, it should be noted that these stress differences related to orbit position and size proportion are quantitatively low. The differences of Von Mises stress in the defined points of the skull (S3 and S4 Tables) reveal that most of the areas are not suffering important variations when variables h and S are changed. In general, only the Von Mises stress recorded in Pineal Foramen (PF), Cultriform Process (CP) and at the triple suture joint of the squamosal, supratemporal and postorbital (SSP) present important differences for the bilateral bite when the orbits are placed on a posterior position: very close to the Pineal Foramen (the differences increase up to 20%). We want to emphasise the values obtained in the Pineal Foramen (PF) that can rise up to the 50% when the position of the orbits are close to the posterior part of the skull. When the orbits are far from the Pineal Foramen (PF), in the central area of the vomer (CV) and the posterior part of the cultriform process (CP), the percent differences increase up to 10%. When the orbits are placed on the maximum posterior extreme case (h = 2.5), the results obtained around the middle point of the Parasphenoid (PPH) and the joint point between the parietals and postparietals (PPP), the stress increases only up to 5%. According to S3 and S4 Tables, the changes in the values obtained in the middle area along the nasals suture (NS), the joint between the parietals and the postparietals (PPP) and the middle point of the parasphenoid (PPH) are always less than a 5% of the value for the original shape (h = 17.5 mm and S = 1).

#### Correlation

**Fig 9:**
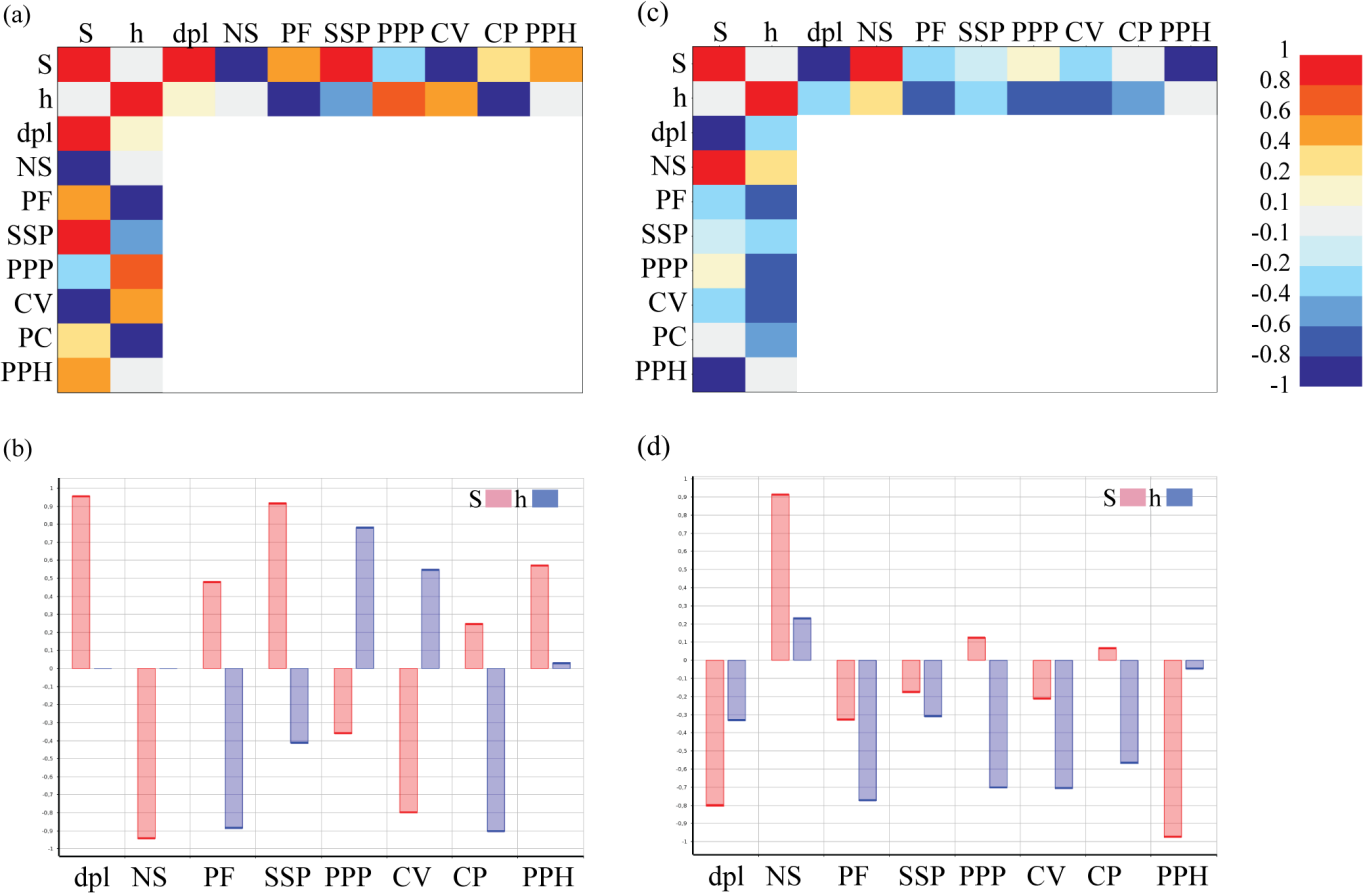
Spearman rank correlation coefficient between size of the orbit (S), position of the orbit (h) and stress levels recorded at the selected points. The maximum displacement is also recorded (dpl).(a) and (b) values for the bilateral case, while (c) and (d) are the values for the skull raising case.

During bilateral bite, all the regressions with the size proportion of the orbit (S) were significant (S9 Table). Despite this, the maximum displacement, the triple suture joint of the squamosal, supratemporal and postorbital (SSP) and the cultriform process (CP), have really low slopes, indicating that, although correlated, changes in the orbit size have a small impact on the changes in stress values. On the other hand, the joint point between the parietals and postparietals (PPP) and the middle point of the parasphenoid (PPH) have larger positive values, meaning that larger orbits produce larger stress values at those points, while the opposite occurs at the central area of the vomer (CV) and at the nasals sutures (NS) (stress values are smaller when the orbit increases in size). Finally, the Pineal Foramen (PF) lacked homoscedasticity of residuals, indicating that a linear regression was not an adequate fit for this data (S9 Table). In any case, the Spearman coefficient for this point was low; indicating that if any correlation existed it was not strong (Fig 9). When the position of the orbit is changed (h), three regressions showed heteroscedastic residuals; Cultriform Process (CP), Pineal Foramen (PF) and the maximum displacement (S9 Table). For the Cultriform process (CP) and the Pineal Foramen (PF), however, the Spearman correlation is high and negative (Fig 9). Despite all having a significant relationship, only two points have a slope larger than 0.01/-0.01, indicating variation in the stress with the parameterization of the orbit: the joint point between the parietals and postparietals (PPP) and the central area of the vomer (CV). It is worth mentioning that those slopes are of the order of ten times smaller than the larger slopes when the size of the orbit was modified (S9 Table). On the other hand, the nasals suture (NS) showed a non-significant relationship.

In relation with Spearman coefficient (Fig 9), the results point out that changes on the orbit size (S) are strongly positively correlated (although not necessarily in a linear manner) with the triple suture joint of the squamosal, supratemporal and postorbital (SSP) and the displacement (i.e. larger orbits were associated with increased stress levels) and correlated, but to a lesser extent, with the Pineal Foramen (PF) and the middle point of the parasphenoid (PPH). On the other hand, the size of the orbit is negatively correlated (i.e. larger orbits were associated with reduced stress levels) with the nasals suture (NS) and central area of the vomer (CV). Changes in the position of the orbit (h) affected the stress levels in an opposite manner, having a high negative correlation with the Pineal Foramen (PF) and the Cultriform Process (CP), while the positive correlations are with the joint point between the parietals and postparietals (PPP) and the central area of the vomer (CV).

#### Multivariate Analyses

**Fig 10:**
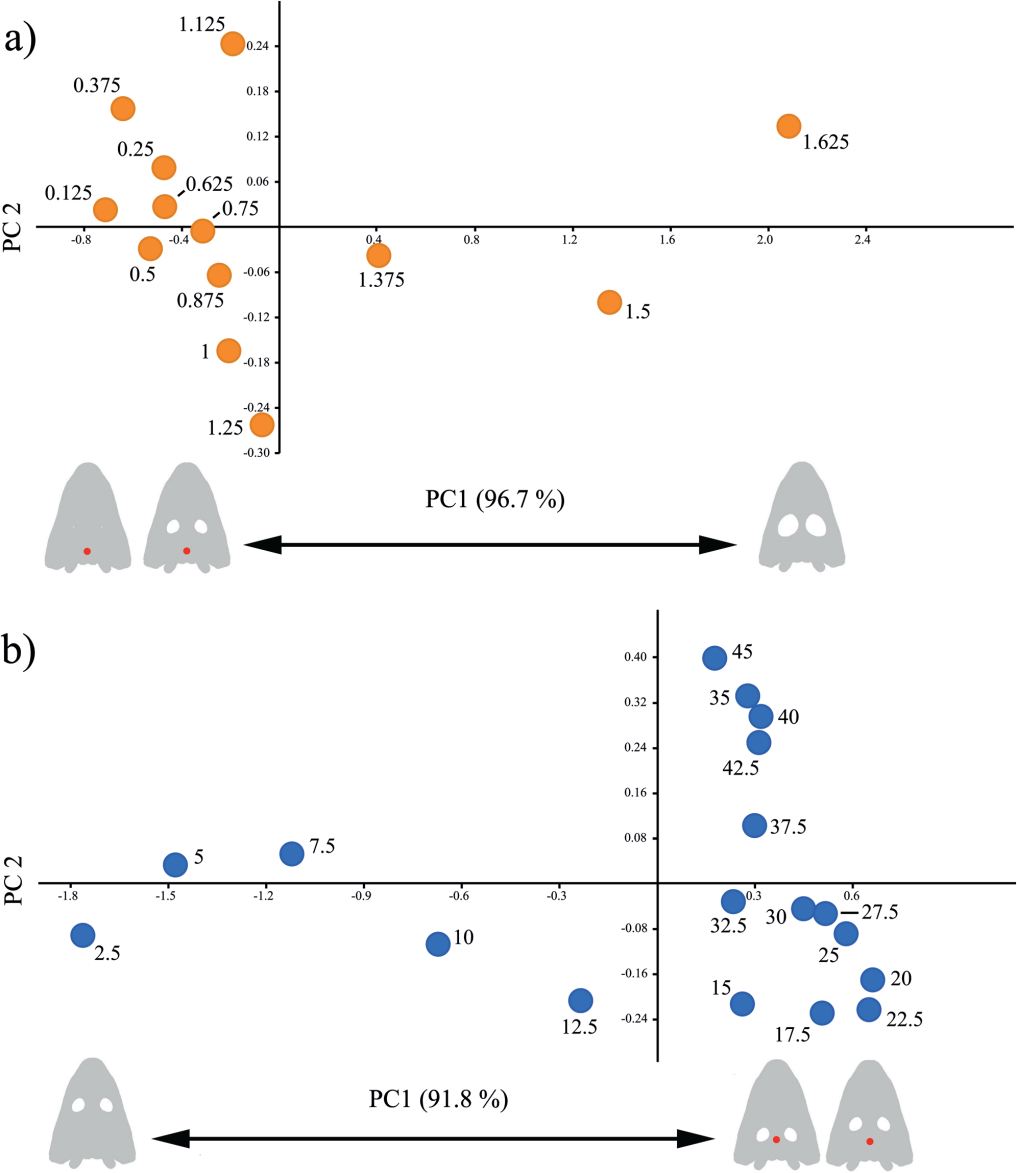
Dispersion plot of the first two PCs of the PCAs for the bilateral case. Red dots on the skulls drawing indicates areas with high stress according to the first PCA (i.e. variables with higher loading). a) Orbit size. b) Orbit location.

Taking the orbit size (S) into consideration, the first PC explains almost all the variance (S10 Table). The Pineal Foramen (PF) is the variable that contributes the most to the PC1 (S11 Table), therefore this axis separates orbits of small and medium size from really large orbits (1.375 onwards, Fig 10(A)), with the latter having high stress at the pineal foramen. This pattern is repeated by the orbit position, with the first PC explaining more than 90% of the variance (S10 Table), and the pineal foramen being responsible of most of the variation in this axis (S11 Table). In this case, the stress is much reduced when the orbit is positioned at the anterior part of the skull, but values intermediate and posterior located have equally high stress. A small part of the variance (6.7%) can be attributed to the central area of the vomer (CV) with values larger than 40 having a big stress in that area (Fig 10(B)).

### Skull-raising system

#### FEA and PA

The value of the maximum displacement is uniform and slightly varies when the position and the size of the orbits changes (less than 2%, see S7 and S8 Tables) and, as in the bilateral bite, the position of the maximum displacement is also the same when the size of the orbits is varied. The maximum displacement is found on the snout, especially on its anterior part, behaving as a cantilever beam during bending.

Stress peaks in the posterior part of the palate, in particular on the parasphenoid and exoccipitals. Less important loadings accrue in the cultriform process (CP), and to the central part of the pterygoids. Considering parameterization, peak values always occur in the same areas not depending on the size and the position of the orbits. Observing the numerical values recorded for the Von Mises stress in the different points of the skull (S5 and S6 Tables) and the percent differences (S7 and S8 Tables), we noted that, most of these points not suffered important variations when the parameters h (orbit position) and S (orbit size proportion) were changed. The Von Mises stress recorded only presented significant changes in the Pineal foramen (PF) and in the triple suture joint of the squamosal, supratemporal and postorbital (SSP) for the skull-raising system when the orbits are placed in an extreme position, fairly close to the pineal foramen. According to S7 and S8 Tables, the differences obtained in most of the points are always less than a 5% of the value for the original shape (h = 18 mm and S = 1) whereas the values obtained in the Pineal foramen (PF) can rise up to 60% when the position of the orbits is pushed far backward. In the same way, the values in the triple suture joint of the squamosal, supratemporal and postorbital (SSP) rise to 30% when the position of the orbits is similarly placed to the back. Interestingly, the results also reveal (Fig 6) that when the size proportion of the orbits (S) is bigger or when the orbits are extremly anteriorly positioned, a notable stress concentration appears in the interorbital region. However, this stress concentration is far from the higher stress values present on the parasphenoid and exoccipitals.

#### Correlation

Regarding the skull raising system, when S was changed, linear regressions with the triple suture joint of the squamosal, supratemporal and postorbital (SSP) turn out to be non-significant. Regardless of the significant results, the slopes for all the variables are so small that do not produce an effective change in the stress results, and the same happens when it is the position of the orbit what changes.

Analysing the Spearman correlation coefficient (Fig 9), the nasals suture (NS) is the only variable that shows larger stress values when increasing the size or the position of the orbits. Otherwise, the middle point of the parasphenoid (PPH) and the maximum displacement (dpl) have a high negative relationship with orbit size, whereas the Pineal foramen (PF), the joint suture between the parietals and postparietals (PPP), the central area of the vomer (CV) and the cultriform process (CP) are those more correlated (negatively) with the position of the orbits.

#### Multivariate analyses

**Fig 11:**
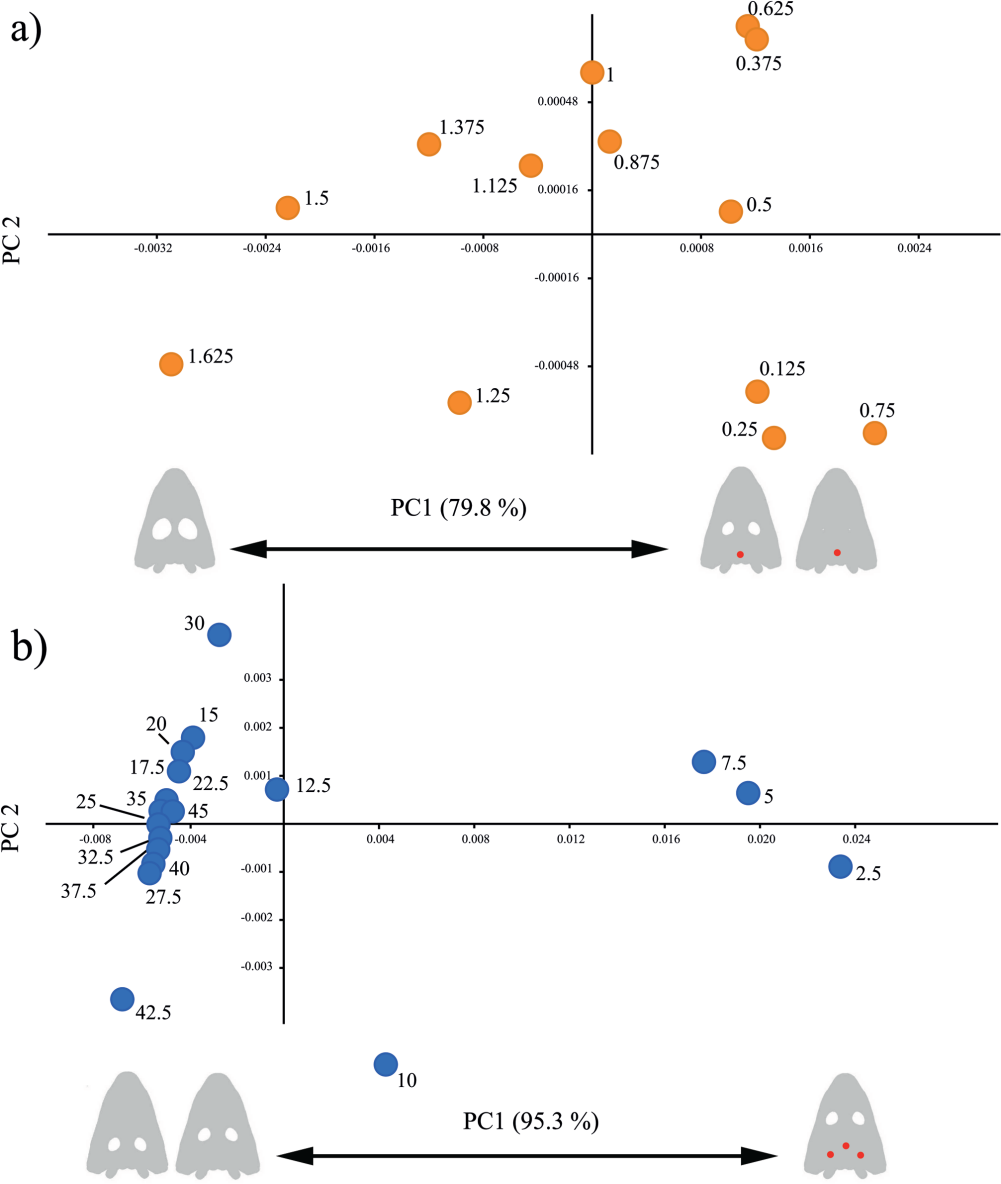
Dispersion plot of the first two PCs of the PCAs for the skull raising case. Red dots on the skulls drawing indicates areas with high stress according to the first PCA (i.e. variables with higher loading). a) Orbit size without pineal foramen data. b) Orbit location.

For the size of the orbit, the pattern is not clear, as a large part of the variance is accounted by one case with high stress at the pineal foramen. If repeating the PC without the pineal foramen stress data, almost 80% of the variance is due to high stress in the parasphenoid when the orbit grows smaller (Fig 11(A)). Regarding the location of the orbit, the PCA shows that orbits located frontally (10 and smaller, Fig 11(B)) have a high stress in the supratemporal area, and to a lesser extent, in the pineal foramen (S11 Table). That accounts for more than 95% of variance (S10 Table).

## Discussion

Early amphibians, and in particular Temnospondyls, had different lifestyles occupying several ecological niches in aquatic, semi aquatic and terrestrial environments. The diversity of temnospondyls varied over time since their origin in the Carboniferous, but of special interest is the increased diversity just after the End-Permian mass extinction [41,42]. In particular, Stereospondyls radiated after this event, possibly in relation to the extinction of basal archegosauriforms [43]. The skull evolution from the ancestral semi-aquatic stem-stereospondyls such as *Capetus* and *Palatinerpeton*, with short tooth-rows and fang pairs on the vomers, palatines and ectopterygoids [19], varied in the different clades of stereospondyls. Orbit position and size were two of the most variable morphological characters.

FEA and PA analysis developed in this work revealed that changes in the size and position of the orbit in most cases did not affect stress distribution during bilateral biting. The highest stresses were found around the otic notch and posterior part of the skull. In the case of skull raising, stress distribution was independent of changes in orbit size or position. In this case, the stress concentrated on the posterior part of the skull (e.g. parasphenoid) and occipital area (e.g. exoccipital) but also with lesser importance in the cultriform area. On the other hand, when there is a relationship between the orbit size and location and the stress pattern (Figs 10 and 11), it affects non vital areas such as the pineal foramen. All these results strongly support the hypothesis of an important mechanical plasticity in the stereospondyl skull, which allowed them to achieve their ecomorphological diversity, especially during the Triassic period, in clades such as lydekkerinids, trematosaurs, capitosaurs or metoposaurs. More important, considering the constructional morphology model [44], the results obtained in this work open the possibility to shed new light on the historical, functional and structural constraints of these early amphibians.

Stereospondyls probably used a bilateral bite due to the absence of a secondary palate and were less optimized to resist both bending and torsional forces during biting than other vertebrates [9]. Regarding taxa with oversized orbits, it should be considered that these oversized orbits were only found in in gigantic sized stereospondyls (e.g. *Mastodonsaurus*, *Eryosuchus*). In many vertebrates, large taxa tend to have larger bite forces due to muscle and jaw size. In Stereospondyls the possession of big orbits may be related to gigantism as in these forms the stress patterns do not affect (or slightly affect) the feeding strategy of these predators due to their size. However, future analysis should include gigantic specimens to assess this issue with confidence. Considering the position of the orbits, its ancestral position was probably similar to basal capitosaurs (as *E*. *madagascariensis*), as it is known for actinodontids and rhinesuchids. In agreement with FEA results, changes in orbit position slightly affect the results, but may reflect that a posterior positioning of the orbits was less optimal due to the stress suffered in the braincase region and some palate structures (such as the cultriform process). On the contrary, its positioning to a more anterior placement, as slightly present in trematosaurs or in extreme case in metoposaurs, reduced stress distribution in the braincase region although increased it in the interorbital region. Moreover, the results suggest that the great diversity of orbit characters in Stereospondyls could only be constrained when the orbits were extremely posterior positioned (as the stress increases) and is in agreement with the fossil record, as to date no taxa is known with these extreme morphological character. In a similar way, oversized orbits could be constrained but may be related to other features (as gigantism) for the viability of this character, as also found in the fossil record.

Trematosaurs were most probably active fish eaters found in fluvial but also brackish environments and possibly their orbit position allowed better catching precision of prey. On the other hand, metoposaurs may obtain an ecological and niche differentiation in the Late Triassic ecosystems (with usual presence of the broad snout cyclotosaur capitosaurids and the slender-snouted phytosaurs) due to the mechanical plasticity of the stereospondyli skull with posteriorly positioned orbits with few (or no) structural consequences. During skull raising, stress concentrated at the most posterior part of the skull, interestingly also affecting the cultriform process, although less importantly. The presence of low levels of stress in the cultriform process during the skull raising while no stress is present in this structure during biting was of particular interest because these amphibians did not present a secondary palate (as present in archosaurs with important biomechanical implications). This is indicative of the biomechanical role of the cultriform process during feeding and raising skull, and future studies are required to analyse this structure. The morphology and thickness of the cultriform process varies throughout the different clades of temnospondyls, and future studies on different clades should analyse the biomechanical role of this structure.

Considering the parameterization during skull raising, possessing big sized orbits or having them in an anterior position implied an increased stress on the interorbital region. However, the correlation between orbit size and the stress found in the nasal region could partially explain the nares sizes and morphology, as well as the presence of sympheseal tusks, to decrease and to dissipate the stress generated during skull raising. Similarly, the correlation between orbit size and stress found in the parasphenoid region suggest that big sized orbits might reduce stress on the braincase region.

After the End-Permian mass extinction, the freshwater terrestrial ecosystems were re-conquered by different survivor groups. During the Early Triassic, trematosaurs replaced the Permian archegosaurs with similar ecomorphological appearance and antero-positioned orbits probably as active fish hunters living in fluvial and brackish environments [9]. This group decreased during the Middle Triassic, being relictual until the Late Triassic-Jurassic [45,46]. Other Stereospondyls were present also in the Early Triassic terrestrial environments, such as the rhytidosteideids, lydekkerinids and capitosaurs, being aquatic or semi-aquatic animals. Lydekkerinids and capitosaurs were probably also active predators with a probably wider range of prey in comparison with trematosaurs. Of special interest, capitosaurs radiated specially during the Middle Triassic acquiring some taxa gigantic sizes with the presence of taxa with big sized orbits (such as *Eryosuchus* or *Mastodonsaurus*). Interestingly, during the late Middle Triassic the first members of the metoposaurid group appeared as the case of *Callistomordax kugleri* from the late Ladinian of Germany. Metoposaurs were members of the trematosauroid clade (see [47] for discussion) and presented antero-positioned orbits with a broad snout morphology in comparison with the slender snouts of trematosaurs. Metoposaurs radiated especially during the Late Triassic with extreme anteropositioned orbits as found in *Apachesaurus*, *Dutuitosaurus*, *Metoposaurus* or *Buettneria* from Europe, Africa and North America [19]. Other broad-snouted stereospondyls typically also lived in these Late Triassic terrestrial ecosystems, with special dominance of the capitosaur genus *Cyclotosaurus*, while the slender-snouted animals from these ecosystems were the archosaur group of phytosaurs. In this ecosystem scheme, the ecomorphological disparity between the broad-snouted stereospondyls and the slender-snouted phytosaurs is very clear, while within the broad-snouted stereospondyls the orbit characters, in particular orbit position, played a key role in the ecology of these two predators; while capitosaur taxa were most probably active predators for a significant range of prey, the metoposaurs were probably less active and sit-wait predators. At the end of the Late Triassic, this ecosystem scheme changed due to the radiation and dominance of some neosuchian groups replacing the stereospondyls causing the dissapareance or decrease of these anamniotes.

## Conclusion

In conclusion, the characterization of the orbit parameters enabled to explore their biomechanical role but portioning the ecological niche of different Stereospondyls groups. Joining FEA and PA to analyse orbits parameters reveal that under bilateral biting and skull raising the mechanical capabilities of these predators did not suffer many changes. In the light of the results, orbit position and size did not constrain the diversity of the group and the great plasticity known for fossil record of Stereospondyls could be at least partially explained by the little influence of orbit size or position in most cases. In extreme cases, the possession of very big sized orbits is potentially related to acquiring gigantic body sizes in these taxa. In the case of extremely anterior positioned orbits, these structural changes imply a reduction of stress to the braincase region. During the Triassic period, different groups as trematosaurs, lyddekerinids, capitosaurs or metoposaurs evolved taking advantage of the empty niches after the End- Permian mass extinction and diversified in the terrestrial environments thanks to the great plasticity as demonstrated for the orbit characters, with an important role in the ecological niche differentiation and feeding habits.

The mixing of PA and FEA to evaluate mechanical capabilities of biological structures is novel in vertebrate palaeontology and the results herein reported demonstrate its potential to reproduce these analyses in other enigmatic or complex structures that could be analysed with these techniques.

## Supporting Information

S1 Document Testing the influence of Adductor Mandibulae Internus (AMI). (DOCX)

S2 Document Testing the influence of the slenderness. (DOCX)

S1 Fig Loadings and boundary conditions on the skull model for the bilateral bite in both extreme cases with AMI (S = 1.625 and h = 2.5 mm). (TIF)

S2 Fig Displacements (mm) and equivalent Von Mises stresses (MPa) in bilateral bite for (a) (b) h = 2.5 mm and (c) (d) S = 1.625 with the inclusion of AMI and in bilateral bite for (e) (f) h = 2.5 mm and (g) (h) S = 1.625 without the inclusion of AMI. (TIF)

S3 Fig Displacements (mm) and equivalent Von Mises stresses (MPa) in bilateral bite for (a) (b) h = 2.5 mm, h = 17.5 mm and h = 40 mm and (c) (d) S = 0.125, S = 1 and S = 1.625 for the long-slender snouted morphotype. (TIF)

S4 Fig Displacements (mm) and equivalent Von Mises stresses (MPa) under a skull raising loading for (a) (b) h = 2.5 mm, h = 17.5 mm and h = 40 mm and (c) (d) S = 0.125, S = 1 and S = 1.625 for the long-slender snouted morphotype. (TIF)

S5 Fig Displacements (mm) and equivalent Von Mises stresses (MPa) in bilateral bite for (a) (b) h = 2.5 mm, h = 17.5 mm and h = 40 mm and (c) (d) S = 0.125, S = 1 and S = 1.625 for the broad skull and short-snouted morphotype. (TIF)

S6 Fig Displacements (mm) and equivalent Von Mises stresses (MPa) under a skull raising loading for (a) (b) h = 2.5 mm, h = 17.5 mm and h = 40 mm and (c) (d) S = 0.125, S = 1 and S = 1.625 for the broad skull and short-snouted morphotype. (TIF)

S1 Table Von Mises stress and displacements obtained for the parameterization of the position of the orbits (h) under a bilateral bite. (DOCX)

S2 Table Von Mises stress and displacements obtained for the parameterization of the size of the orbit (S) under a bilateral bite. (DOCX)

S3 Table Percent differences of Von Mises stress and displacements obtained for the parameterization of the position of the orbits (h) under a bilateral bite in relationship its original position (h = 17.5 mm). (DOCX)

S4 Table Percent differences of Von Mises stress and displacements obtained for the parameterization of the size of the orbits (S) under a bilateral bite in relationship with the original size of the orbits (S = 1). (DOCX)

S5 Table Von Mises stress and displacements obtained for the parameterization of the position of the orbit (h) during the skull-raising loading. (DOCX)

S6 Table Von Mises stress and displacements obtained for the parameterization of the size of the orbit (S) during the skull-raising loading. (DOCX)

S7 Table Percent differences of Von Mises Stress and displacements obtained for the parameterization of the position of the orbits (h) during the skull-raising loading in relationship with its original position (h = 17.5 mm). (DOCX)

S8 Table Percent differences of Von Mises Stress and displacements obtained for the parameterization of the size of the orbit (S) during the skull-raising loading in relationship with the original size of the orbits (S = 1). (DOCX)

S9 Table Statistics of the OLS regression between orbit size and location and the stress variables and displacement. (DOCX)

S10 Table Percent of explained variable for each PC at the different PCAs developed in this work. (DOCX)

S11 Table Loadings of the original variables for each PC at the different PCAs developed in this work. (DOCX)

## References

[1] Zienkiewicz O. The Finite Element Method in Engineering Science Finite Element Methods In Engineering Science. McGraw-Hill; 1971 pp. 98–359.

[2] Rayfield EJ. Finite Element Analysis and Understanding the Biomechanics and Evolution of Living and Fossil Organisms. Annu Rev Earth Planet Sci. 2007;35: 541–576. 10.1146/annurev.earth.35.031306.140104

[3] Cox PG, Fagan MJ, Rayfield EJ, Jeffery N. Finite element modelling of squirrel, guinea pig and rat skulls: using geometric morphometrics to assess sensitivity. J Anat. 2011;219: 696–709. 10.1111/j.1469-7580.2011.01436.x 21974720 PMC3237878

[4] McHenry CR, Wroe S, Clausen PD, Moreno K, Cunningham E. Supermodeled sabercat, predatory behavior in Smilodon fatalis revealed by high-resolution 3D computer simulation. Proc Natl Acad Sci U S A. 2007;104: 16010–5. 10.1073/pnas.0706086104 17911253 PMC2042153

[5] Kupczik K, Dobson CA, Fagan MJ, Crompton RH, Oxnard CE, O’Higgins P. Assessing mechanical function of the zygomatic region in macaques: validation and sensitivity testing of finite element models. J Anat. 2007;210: 41–53. 10.1111/j.1469-7580.2006.00662.x 17229282 PMC2100262

[6] Tseng ZJ, McNitt-Gray JL, Flashner H, Wang X, Enciso R. Model Sensitivity and Use of the Comparative Finite Element Method in Mammalian Jaw Mechanics: Mandible Performance in the Gray Wolf. PLoS One. 2011;6: 12 10.1371/journal.pone.0019171 PMC3084775 21559475

[7] Walmsley CW, McCurry MR, Clausen PD, McHenry CR. Beware the black box: investigating the sensitivity of FEA simulations to modelling factors in comparative biomechanics. PeerJ. 2013;1: e204 10.7717/peerj.204 24255817 PMC3828634

[8] Piras P, Maiorino L, Teresi L, Meloro C, Lucci F, Kotsakis T, et al Bite of the cats: relationships between functional integration and mechanical performance as revealed by mandible geometry. Syst Biol. 2013;62: 878–900. 10.1093/sysbio/syt053 23925509

[9] Fortuny J, Marcé-Nogué J, De Esteban-Trivigno S, Gil L, Galobart À. Temnospondyli bite club: ecomorphological patterns of the most diverse group of early tetrapods. J Evol Biol. 2011;24: 2040–54. 10.1111/j.1420-9101.2011.02338.x 21707813

[10] Fortuny J, Marcé-Nogué J, Gil L, Galobart À. Skull mechanics and the evolutionary patterns of the otic notch closure in capitosaurs (Amphibia: Temnospondyli). Anat Rec (Hoboken). 2012;295: 1134–46. 10.1002/ar.22486 22573567

[11] Warren A. Secondarily aquatic temnospondyls of the Upper Permian and Mesozoic In: Carroll HH& RL, editor. Amphibian Biology. Beatty & Sons; 2000 pp. 1121–1149.

[12] Schoch RR. Life cycles, plasticity and palaeoecology in temnospondyl amphibians. Smith A, editor. Palaeontology. 2014;57: 517–529. 10.1111/pala.12100

[13] Schoch RR. Amphibian Evolution: The Life of Early Land Vertebrates New Jersey: Wiley-Blackwell; 2014.

[14] Witzmann F. Cranial morphology and ontogeny of the Permo-Carboniferous temnospondyl Archegosaurus decheni Goldfuss, 1847 from the Saar–Nahe Basin, Germany. Trans R Soc Edinb Earth Sci. 2006;96: 131–162. 10.1017/S0263593300001279

[15] Erickson GM, Gignac PM, Steppan SJ, Lappin AK, Vliet KA, Brueggen JD, et al Insights into the ecology and evolutionary success of crocodilians revealed through bite-force and tooth-pressure experimentation. PLoS One. 2012;7: e31781 10.1371/journal.pone.0031781 22431965 PMC3303775

[16] Walmsley CW, Smits PD, Quayle MR, McCurry MR, Richards HS, Oldfield CC, et al Why the Long Face? The Mechanics of Mandibular Symphysis Proportions in Crocodiles. PLoS One. 2013;8: e53873 10.1371/journal.pone.0053873 23342027 PMC3547052

[17] Piras P, Buscalioni AD, Teresi L, Raia P, Sansalone G, Kotsakis T, et al Morphological integration and functional modularity in the crocodilian skull. Integr Zool. 2014;9: 498–516. 10.1111/1749-4877.12062 25236418

[18] Busbey AB. The structural consequences of skull flattening in crocodilians In: Thomason JJ, editor. Functional Morphology in Vertebrate Paleontology. Cambridge: Cambridge University Press; 1995 pp. 173–192.

[19] Schoch RR, Milner A. Stereospondyli In: Wellnhofer P, editor. Encyclopedia of Paleoherpetology 3B. München: Dr. Friedrich Pfiel; 2000 pp. 1–204.

[20] Steyer JS. A revision of the early Triassic “capitosaurs” (Stegocephali, Stereospondyli) from Madagascar, with remarks on their comparative ontogeny. Journal of Vertebrate Paleontology. 2003. pp. 544–555. 10.1671/1740

[21] Fortuny J, Galobart À, De Santisteban C. A New Capitosaur from the Middle Triassic of Spain and the Relationships within the Capitosauria. Acta Palaeontol Pol. 2011;56: 553–566. 10.4202/app.2010.0025

[22] Maganuco S, Steyer JS, Pasini G, Bénéteau M, Lorrain S, Bénéteau A, et al An exquisite specimen of “Edingerella Madagascariensis”(Temnospondyli) from the Lower Triassic of NW Madagascar: Cranial Anatomy, Phylogeny, and Restorations. Mem Soc Ital Sci Nat Mus Civ Stor Nat Milano. 2009;XXXVI: 1–72.

[23] Marcé-Nogué J, Fortuny J, Gil L, Galobart À. Using Reverse Engineering to Reconstruct Tetrapod Skulls and Analyse its Feeding Behaviour Proc Thirteen Int Conf Civil, Struct Environ Eng Comput. Civil-Comp Press; 2011;

[24] Schoch RR. The neurocranium of the stereospondyl Mastodonsaurus giganteus. Palaeontology. 2002;45: 627–645.

[25] Dutuit J-M. Introduction à l’étude paléontologique du Trias continental marocain : description des premiers Stégocéphales recueillis dans le couloir d'Argana (Atlas occidental) Paris: Éditions du Muséum; 1976.

[26] Bystrow AP, Efremov JA. Benthosuchus sushkini Efr., a labyrinthodont from the Eotriassic of Sharzhenga River. Trav l’Institut Paleontol Acad des Sci l'URSS. 1940;10: 1–152.

[27] Carroll R, Holmes R. The skull and jaw musculature as guides to the ancestry of salamanders. Zool J Linn Soc. 1980;

[28] Witzmann F, Schoch RR. Reconstruction of cranial and hyobranchial muscles in the Triassic temnospondyl Gerrothorax provides evidence for akinetic suction feeding. J Morphol. 2013;274: 525–42. 10.1002/jmor.20113 23280767

[29] Alexander RM. Exploring biomechanics animals in motion R. McNeill Alexander. New York Scientific American Library Distributed by W.H. Freeman; 1992.

[30] Gil L, Marcé-Nogué J, Sánchez M. Insights into the controversy over materials data for the comparison of biomechanical performance in vertebrates. Palaeontol Electron. 2015; in press.

[31] Currey JD. The evolution of the mechanical properties of amniote bone. J Biomech. Elsevier; 1987;20: 1035–1044. 10.1016/0021-9290(87)90021-2 3429455

[32] Marcé-Nogué J, Fortuny J, Gil L, Sánchez M. Improving mesh generation in Finite Element Analysis for functional morphology approaches. Spanish J Palaeontol. 2015;in press.

[33] Damiani RJ. A systematic revision and phylogenetic analysis of Triassic mastodonsauroids (Temnospondyli: Stereospondyli). Zool J Linn Soc. 2001; 379–482. 10.1006/zjls.2001.0304

[34] Sulej T, Majer D. The temnospondyl amphibian Cyclotosaurus from the Upper Triassic of Poland. Palaeontology. 2005;48: 157–170.

[35] Steyer JS. The first articulated trematosaur “amphibian”from the Lower Triassic of Madagascar: implications for the phylogeny of the group. Palaeontology. 2002;45: 771–793.

[36] Schoch RR. Comparitive osteology of Mastodonsaurus giganteus from the Middle Triassic of Germany. Stuttgarter Beiträge zur Naturkunde, Stuttgart. 1999; 1–175.

[37] Doblaré M, García JM, Gómez MJ. Modelling bone tissue fracture and healing: a review*1. Eng Fract Mech. 2004;71: 1809–1840. 10.1016/j.engfracmech.2003.08.003

[38] Quinn G, Keough M. Experimental design and data analysis for biologists Cambridge Univ Press; 2002.

[39] Hammer Ø, Harper DAT, Ryan PD. Paleontological statistics software package for education and data analysis. Palaeontol Electron. 2001;4: 9–18. 10.1016/j.bcp.2008.05.025

[40] Croxton FE, Cowden DJ, Klein S. Applied General Statistics. Pitman; 1968. p. 650.

[41] Ruta M, Benton MJ. Calibrated diversity, tree topology and the mother of mass extinctions: The lesson of temnospondyls. Palaeontology. 2008;51: 1261–1288. 10.1111/j.1475-4983.2008.00808.x

[42] Benton MJ, Ruta M, Dunhill AM, Sakamoto M. The first half of tetrapod evolution, sampling proxies, and fossil record quality. Palaeogeogr Palaeoclimatol Palaeoecol. 2013;372: 18–41. 10.1016/j.palaeo.2012.09.005

[43] Stayton CT, Ruta M. Gometric orphometrics of the skull roof of Stereospondyls (Amphibia: Temnosponyli). Palaeontology. 2006;49: 307–337. 10.1111/j.1475-4983.2006.00523.x

[44] Seilacher A. Arbeitskonzept zur konstruktions-morphologie. Lethaia. 1970;3: 393–396.

[45] Schoch RR, Milner A, Hellrung H. The last trematosaurid amphibian Hyperokynodon keuperinus revisited. Geol und Paläontologie. 2002; 1–9.

[46] Maisch MW, Matzke AT, Sun G. A relict trematosauroid (Amphibia: Temnospondyli) from the Middle Jurassic of the Junggar Basin (NW China). Naturwissenschaften. 2004;91: 589–593. 10.1007/s00114-004-0569-x 15448923

[47] Schoch RR. A new stereospondyl from the German Middle Triassic, and the origin of the Metoposauridae. Zool J Linn Soc. 2008;152: 79–113. 10.1111/j.1096-3642.2007.00363.x

[48] Sulej T. Osteology, variability, and evolution of Metoposaurus, a temnospondyl from the Late Triassic of Poland. Palaeontol Pol. 2007;64: 29–139.

