# Supplementary material for: 3D Computational Mechanics Elucidate the Evolutionary Implications of Orbit Position and Size Diversity of Early Amphibians
Source: PLoS One. 2015 Jun 24;10(6):e0131320. doi: 10.1371/journal.pone.0131320 (PMC4479603; doi:10.1371/journal.pone.0131320)
Supplement: S1 Document — (DOCX) [file pone.0131320.s001.docx]

Supplementary Information

**Testing the influence of Adductor Mandibulae Internus (AMI)**

## Methods

Under the bilateral loading and the parametric analysis two muscles were considered: the Adductor Mandibulae Externus (AME) and the Adductor Mandibulae Posterior (AMP). The Adductor Mandibulae Internus (AMI) was not considered in the main analysis, but *a priori* tested to evaluate the influence of this muscle on the different results obtained. However, due to the negligible influence of this muscle in the stress results was not included.

In the first analysis including AMI together with AME and AMP, the muscular insertion areas of these muscles were defined in the model in order to apply the forces of the muscular contraction during the prehension/bite following the same steps as in the main cases. The direction of these forces was defined by the line that joins the centroid of the insertion area in the skull with its correspondence in the insertion area of the lower jaw. The muscular contraction pressure (Force per unit area) assumed was 0.3 MPa and was applied at the insertion area of each muscle.

In particular, two extreme cases were analysed with and without AMI to test the influence of this muscle. These two cases represent extreme polarities (big sized and extremely posterior positioned orbits) where AMI is expected to have major influence on the results. These cases are: A) Placing the orbit in a very posterior position, that is when the distance between the centre of the pineal foramen and the centre of the orbit is h=2.5 mm and B) the proportion of the orbit size is extremely high, S=1,625. (See S1 Fig. for the boundary conditions of each case).

As used in all the analyses, elastic, linear and homogeneous material properties were assumed for the bone of *Edingerella madagascariensis*, using the following values: E (Young’s modulus): 6.65 GPa and m (Poisson’s ratio) 0.35. The skull was meshed with an adaptive mesh of hexahedral elements around 450000-500000 nodes. The results of the bilateral bite were obtained from FEA. The coloured maps of the displacements and Von Mises Stress are shown for the skull model (S2 Fig.).

## Results and Discussion

The results obtained from these two extreme cases with and without AMI (See S2 Fig.) under bilateral bite reveals that in all cases the presence of AMI (a,b,c and d) or its exclusion (e, f, g and h) is practically the same. These results demonstrate that AMI had small influence in Stereospondyls, as this muscle didn’t played a key role as happened in extant lissamphibians where AMI is one of the main muscles.
