# Supplementary material for: 3D Computational Mechanics Elucidate the Evolutionary Implications of Orbit Position and Size Diversity of Early Amphibians
Source: PLoS One. 2015 Jun 24;10(6):e0131320. doi: 10.1371/journal.pone.0131320 (PMC4479603; doi:10.1371/journal.pone.0131320)
Supplement: S2 Document — (DOCX) [file pone.0131320.s002.docx]

Supplementary Information

**Testing the influence of the slenderness**

## Methods

The 3D skull model of *Edingerella madagascariensis* was wrapped in order to evaluate the morphological disparity of Stereospondyls. In particular, we modified the original geometry to obtain two models that encompass the most extreme morphological disparity of Stereospondyls: one approaching to the long slender snouted trematosaur morphotype tightening the model, and the other to the broad skull and short-snouted morphotype as found in some stereospondyls such as *Sclerothorax* stretching the model in a transversal direction. These geometrical models were obtained by applying a directional scaling in only one direction of the skull obtaining the two analysed models:

a) Long-slender snouted morphotype

b) Broad skull and short snouted morphotype

These changes in the model geometry were applied using the CAD interface tools of the Finite Element Package ANSYS 14.5 and the Finite Element Analysis was done in the same way that the non-modified skull (original geometry): Two different cases of boundary and loading conditions were evaluated representing a bilateral bite and a raising skull system with the same boundary conditions and forces. Due to the change of volume of the model, the values of the forces were scaled according to [1].

The parametric analysis was also done by modifying the values of the same two variables separately: the position of the orbits along the principal axis of the skull and the size of the orbits.

## Results and Discussion

S3, S4, S5 and S6 Figs. show the patterns of stress and displacement of the analysis under a bilateral bite and skull raising system.

**Bilateral loading**

The bilateral loading in the broad skull morphotype present very similar patterns than previously described for the original model. Nonetheless, the stress values are in general lower than the ones found in the original model under this loading. Regarding the displacements, the higher values are found in the cheek region of the skull, as in the original model. Regarding the Von Mises stress, the higher values are found around the otic notches in all cases, with the same pattern found in the original model of the skull previously described for the parameterization of the orbit position (only found in the skull roof, decreasing on the posterior part of the skull and increasing on the interorbital area when orbits are on an anterior position). Considering the size proportion of the orbit, the changes in size also agrees with the results obtained in the original model, increasing the stress in the posterior part of the skull and slightly in the interorbital region.

On the other hand, the long-slender snouted morphotype shows differences in the displacement patterns being the higher values in the interorbital region in all cases instead than in the cheek region due to the elongation of the skull geometry producing the approach of the displacements to the interorbital region. A similar pattern is found in the Von Mises stress: the values are generally higher than the ones found in the original model with the maximum stress values around the interorbital region instead that in the postorbital region. This is because the stretching of the model reduces the interorbital region driving to an important increase of the stresses. The stress pattern don’t suffer modifications when the position of the orbits are placed in a more anterior position but stresses increases in the interorbital region when the orbits are placed anteriorly, as also found in the original model analysis. In the case of the orbit sizes, no changes are found in the palate while the stress also increases in the interorbital region when the size of the orbit is bigger.

**Skull raising system**

The broad skull morphotype presents similar patterns in the FEA results as in the original model. On this way, the higher displacement values are found in the anterior part of the snout. In the case of the Von Mises stress, the stress values are higher than the ones found in the original model, but have the same stress patterns: the highest stress values are found in the posterior part of the palate with less important values in the cultriform process. The skull roof has low levels of stress found in the posterior and interorbital region. Changes in orbit position from a posterior to anterior position show that the stress slightly decreases, particularly in the interorbital region. Regarding the orbit’s size, increasing the size of the orbit, the stress values slightly increases also around the interorbital region.

Under the same loading, the long-slender snouted morphotype present similar displacement pattern than in the original model. The stress pattern is, as in the broad skull morphotype, very similar to the original model, with maximum stress values around the posterior part of the palate (exoccipitals) with less important values in the parasphenoid. In the skull roof, the stresses are in general low, but present especially in the postorbital and interorbital region. When the orbit position moves from a posterior to an anterior position, the stresses from the skull roof also moves anteriorly but not changing its values, while no stress pattern is found in the palate. Finally, when the orbit’s size is increased, stresses slightly increases in the interorbital region.

The results obtained in these extreme geometrical morphotypes follow the general patterns found in the original geometry suggesting that within the natural variation of the group, the consequences of different orbit size and position are comparable and accordingly could be considered mainly jointly. Interestingly, it should be noted also that in elongated skull morphotypes (as trematosaurs), under bilateral bites, low levels of stress are found in endocranial region. Moreover, in the case of elongated morphotype altogether with anterior positioned orbits or big sized ones, stress peaks around the interorbital region. In the other extreme case, the broad skull morphotype during opening the mouth reveals that the cultriform process present high levels of stress giving a potential explanation of the wider cultriform process found in broad skull stereospondyls taxa such as *Sclerothorax* [2] but may also suggesting a low gape opening in these animals.

**References**

1. Fortuny J, Marcé-Nogué J, Heiss E, Sanchez M, Gil L, Galobart À. (2015) 3D Bite Modeling and Feeding Mechanics of the Largest Living Amphibian, the Chinese Giant Salamander *Andrias davidianus* (Amphibia:Urodela). PLoS ONE 10(4): e0121885. doi:10.1371/journal.pone.0121885*.*

2. Schoch RR, Fastnacht M, Fichter J, Keller T. Anatomy and relationships of the Triassic temnospondyl *Sclerothorax*. Acta Palaeontol Pol. 2007;52: 117–136.
