# Supplementary figures and images for: 3D Computational Mechanics Elucidate the Evolutionary Implications of Orbit Position and Size Diversity of Early Amphibians
Source: PLoS One. 2015 Jun 24;10(6):e0131320. doi: 10.1371/journal.pone.0131320 (PMC4479603; doi:10.1371/journal.pone.0131320)

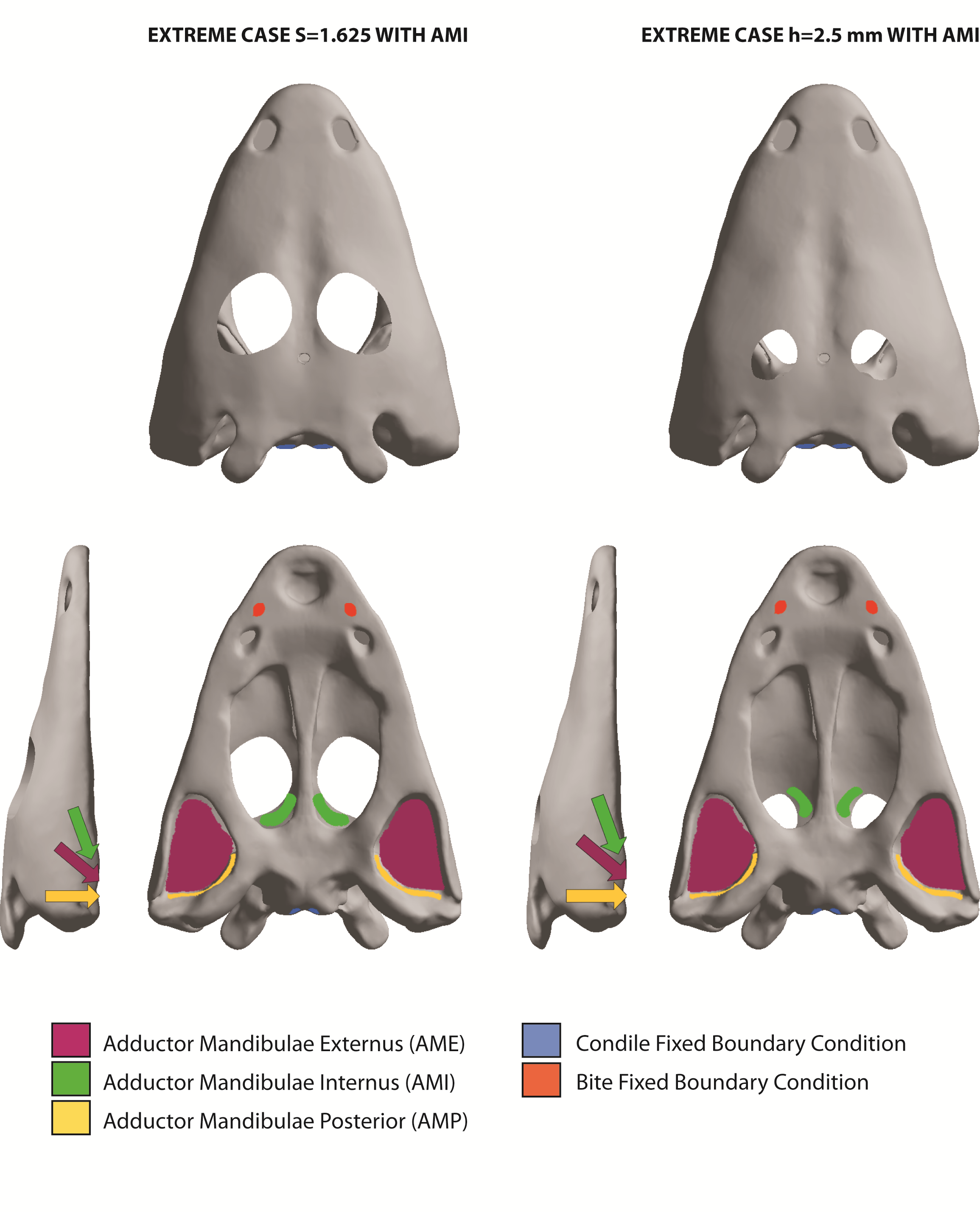

Supplement: S1 Fig — (TIF) [file pone.0131320.s003.tif]

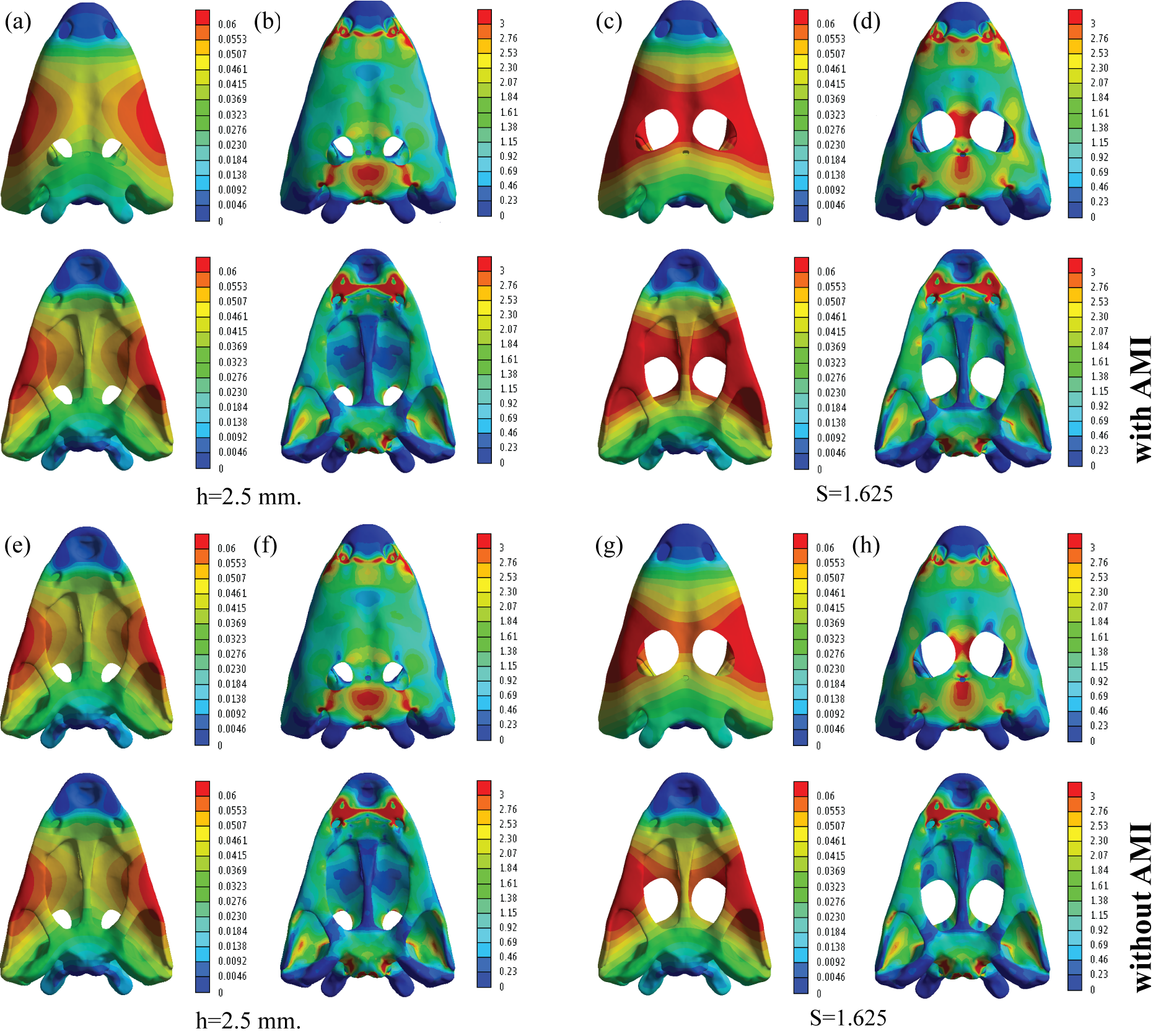

Supplement: S2 Fig — (TIF) [file pone.0131320.s004.tif]

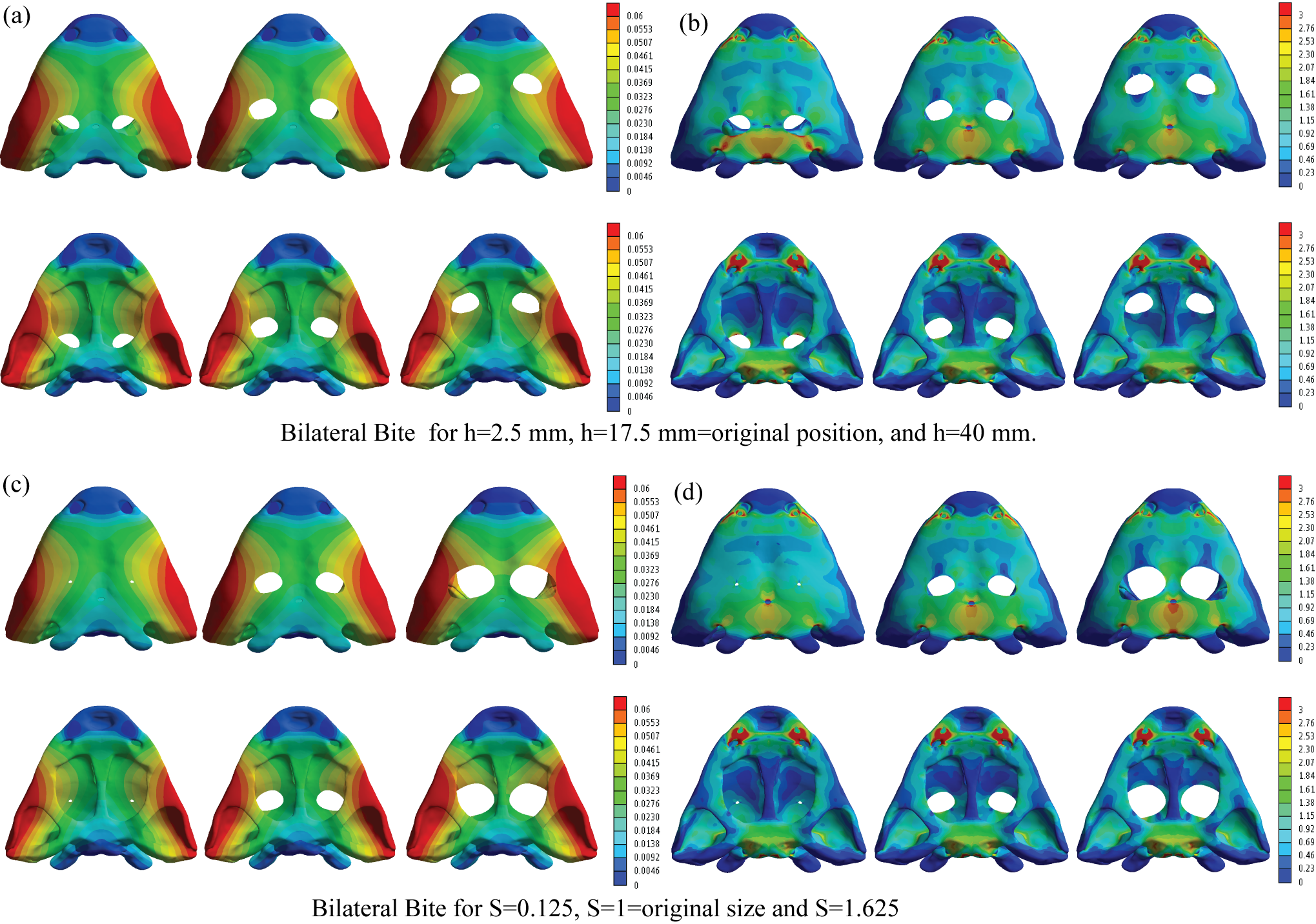

Supplement: S3 Fig — (TIF) [file pone.0131320.s005.tif]

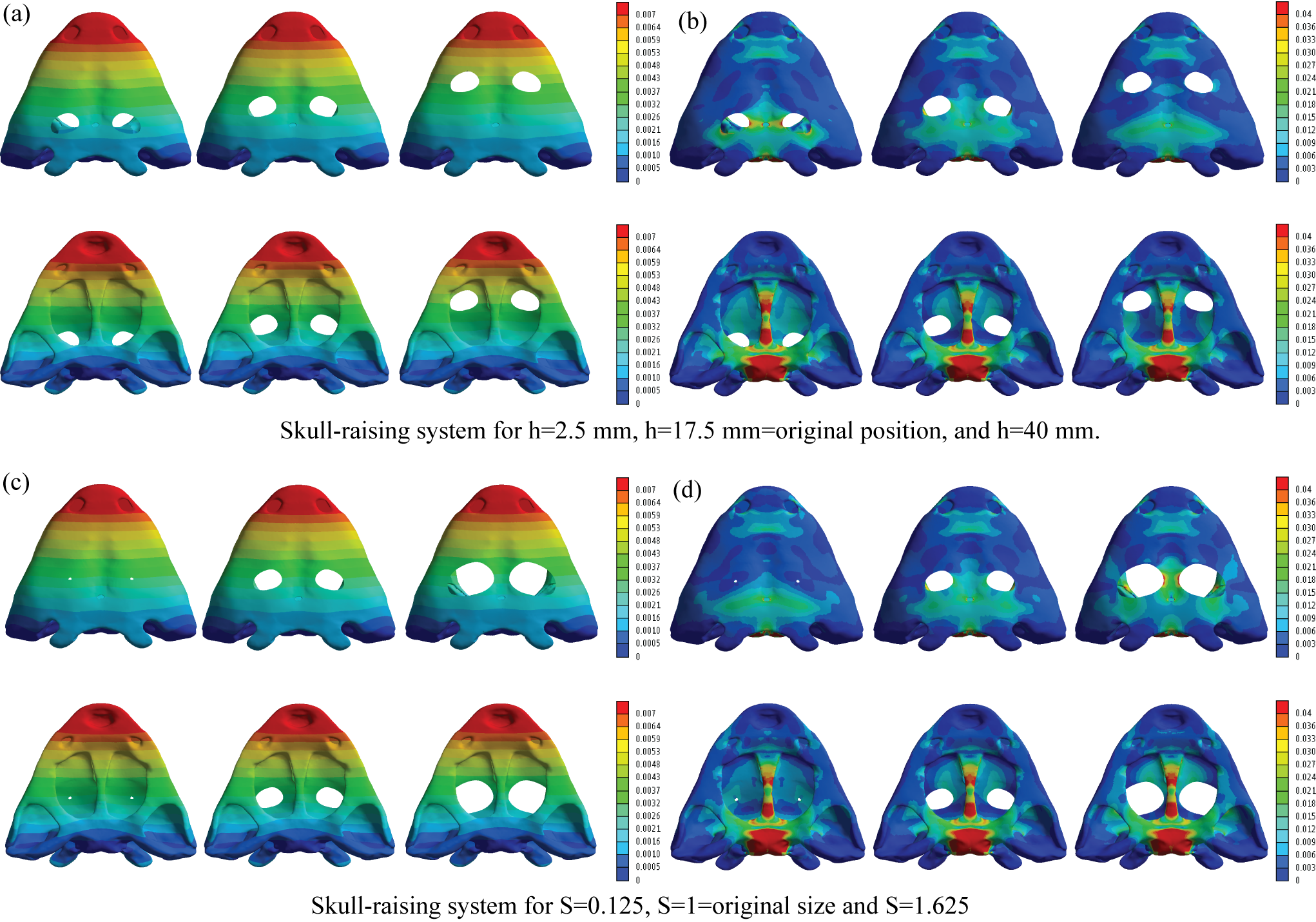

Supplement: S4 Fig — (TIF) [file pone.0131320.s006.tif]

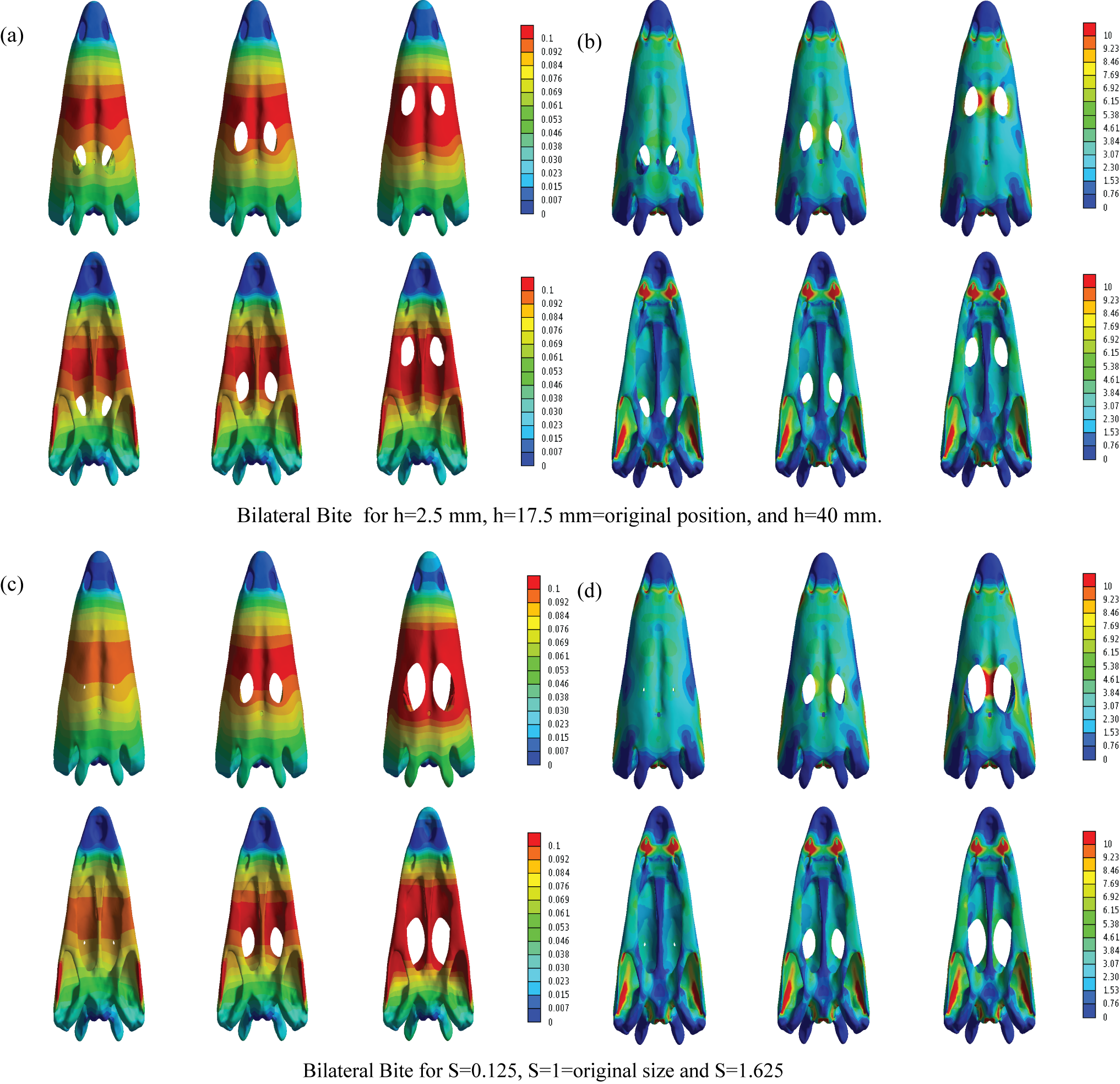

Supplement: S5 Fig — (TIF) [file pone.0131320.s007.tif]

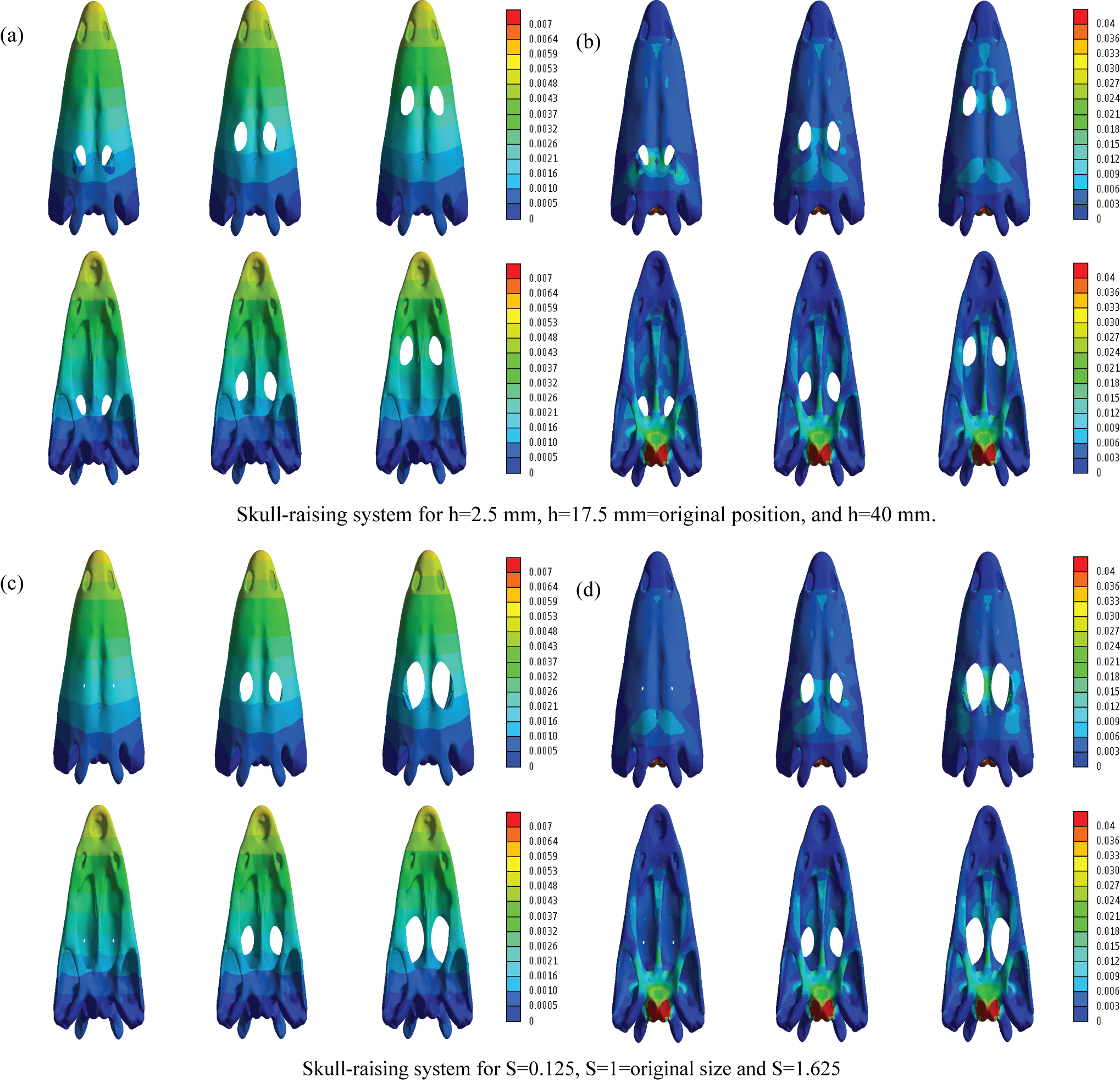

Supplement: S6 Fig — (TIF) [file pone.0131320.s008.tif]
