# Supplementary material for: 3D Computational Mechanics Elucidate the Evolutionary Implications of Orbit Position and Size Diversity of Early Amphibians
Source: PLoS One. 2015 Jun 24;10(6):e0131320. doi: 10.1371/journal.pone.0131320 (PMC4479603; doi:10.1371/journal.pone.0131320)
Supplement: S1 Table — (DOCX) [file pone.0131320.s009.docx]

| Case | h [mm] | NS Von Mises Stress [MPa] | PPP Von Mises Stress [MPa] | PPH Von Mises Stress [MPa] | CV Von Mises Stress [MPa] | CP Von Mises Stress [MPa] | SSP Von Mises Stress [MPa] | PF Von Mises Stress [MPa] | Max. displacement [mm] |
| --- | --- | --- | --- | --- | --- | --- | --- | --- | --- |
| 1 | 2.5 | 3.4797 | 4.1702 | 2.0490 | 3.8599 | 0.2711 | 2.8032 | 3.0104 | 0.0650 |
| 2 | 5 | 3.4811 | 3.8645 | 2.0028 | 3.8887 | 0.2906 | 2.6165 | 3.1914 | 0.0644 |
| 3 | 7.5 | 3.4416 | 3.6889 | 2.0146 | 3.8914 | 0.3219 | 2.4900 | 3.5024 | 0.0640 |
| 4 | 10 | 3.4415 | 3.7739 | 1.9879 | 3.8380 | 0.3422 | 2.4367 | 3.9813 | 0.0639 |
| 5 | 12.5 | 3.5038 | 3.7669 | 1.9970 | 3.8100 | 0.3674 | 2.3564 | 4.4262 | 0.0638 |
| 6 | 15 | 3.4924 | 3.7171 | 1.9667 | 3.8831 | 0.3802 | 2.3110 | 4.9167 | 0.0638 |
| 7 | 17.5 | 3.4770 | 3.6169 | 1.9246 | 3.8717 | 0.3903 | 2.2924 | 5.1463 | 0.0640 |
| 8 | 20 | 3.4378 | 3.6054 | 1.9545 | 3.9643 | 0.3945 | 2.2782 | 5.2969 | 0.0642 |
| 9 | 22.5 | 3.4678 | 3.5777 | 1.8913 | 3.8876 | 0.4046 | 2.2761 | 5.2823 | 0.0646 |
| 10 | 25 | 3.4672 | 3.5326 | 1.9085 | 3.9968 | 0.4085 | 2.2626 | 5.1857 | 0.0649 |
| 11 | 27.5 | 3.4179 | 3.5926 | 1.9055 | 4.0402 | 0.4111 | 2.2520 | 5.1256 | 0.0654 |
| 12 | 30 | 3.4347 | 3.5976 | 1.8955 | 4.0311 | 0.4266 | 2.2448 | 5.0561 | 0.0657 |
| 13 | 32.5 | 3.3910 | 3.6278 | 1.8936 | 4.0083 | 0.4179 | 2.2462 | 4.8445 | 0.0660 |
| 14 | 35 | 3.4379 | 3.6152 | 1.9115 | 4.4099 | 0.4238 | 2.2419 | 4.8393 | 0.0663 |
| 15 | 37.5 | 3.4497 | 3.5140 | 1.9045 | 4.1237 | 0.4388 | 2.2409 | 4.8700 | 0.0663 |
| 16 | 40 | 3.4365 | 3.4387 | 1.9039 | 4.3087 | 0.4417 | 2.2434 | 4.8575 | 0.0664 |
| 17 | 42.5 | 3.4367 | 3.5846 | 1.9027 | 4.3124 | 0.4438 | 2.2423 | 4.8760 | 0.0662 |
| 18 | 45 | 3.4627 | 3.4759 | 1.8837 | 4.4039 | 0.4582 | 2.2427 | 4.7055 | 0.0660 |

**Table S1 Von Mises stress and displacements** obtained for the parameterization of the position of the orbits (h) under a bilateral bite
