# Supplementary material for: 3D Computational Mechanics Elucidate the Evolutionary Implications of Orbit Position and Size Diversity of Early Amphibians
Source: PLoS One. 2015 Jun 24;10(6):e0131320. doi: 10.1371/journal.pone.0131320 (PMC4479603; doi:10.1371/journal.pone.0131320)
Supplement: S2 Table — (DOCX) [file pone.0131320.s010.docx]

| Case | S | NS Von Mises Stress [MPa] | PPP Von Mises Stress [MPa] | PPH Von Mises Stress [MPa] | CV Von Mises Stress [MPa] | CP Von Mises Stress [MPa] | SSP Von Mises Stress [MPa] | PF Von Mises Stress [MPa] | Max. displacement [mm] |
| --- | --- | --- | --- | --- | --- | --- | --- | --- | --- |
| 1 | 0.125 | 3.5784 | 3.5496 | 1.8747. | 4.0548. | 0.4292. | 2.2417. | 4.7281. | 0.0608. |
| 2 | 0.25 | 3.5617 | 3.5160 | 1.8912. | 4.0591. | 0.4260. | 2.2425. | 4.9768. | 0.0609. |
| 3 | 0.375 | 3.5385 | 3.5462 | 1.8607. | 4.1734. | 0.4206. | 2.2498. | 4.8239. | 0.0612. |
| 4 | 0.5 | 3.5151 | 3.4664 | 1.8737. | 3.9664. | 0.4188. | 2.2627. | 4.9017. | 0.0615. |
| 5 | 0.625 | 3.5223 | 3.5772 | 1.8988. | 4.0154. | 0.4120. | 2.2631. | 4.9595. | 0.0619. |
| 6 | 0.75 | 3.4802 | 3.6314 | 1.9373. | 3.9589. | 0.4022. | 2.2662. | 5.0974. | 0.0625. |
| 7 | 0.875 | 3.5012 | 3.6090 | 1.9173. | 3.8830. | 0.4000. | 2.2785. | 5.1570. | 0.0632. |
| 8 | 1 | 3.4710 | 3.6300 | 1.9217. | 3.7803. | 0.3887. | 2.2896. | 5.1699. | 0.0640. |
| 9 | 1.125 | 3.4040 | 3.6490 | 1.9738. | 4.1919. | 0.3812. | 2.3027. | 5.2527. | 0.0650. |
| 10 | 1.25 | 3.3977 | 3.6202 | 2.0009. | 3.6711. | 0.3579. | 2.3136. | 5.2734. | 0.0661. |
| 11 | 1.375 | 3.3531 | 3.6674 | 2.0330. | 3.7984. | 0.3512. | 2.3343. | 5.7816. | 0.0675. |
| 12 | 1.5 | 3.3092 | 3.7583 | 2.0691. | 3.5451. | 0.3366. | 2.3540. | 6.6891. | 0.0694. |
| 13 | 1.625 | 3.2327 | 3.8080 | 2.0460. | 3.6239. | 0.3044. | 2.3767. | 7.4494. | 0.0715. |

**Table S2 Von Mises stress and displacements** obtained for the parameterization of the size of the orbit (S) under a bilateral bite
