# Supplementary material for: 3D Computational Mechanics Elucidate the Evolutionary Implications of Orbit Position and Size Diversity of Early Amphibians
Source: PLoS One. 2015 Jun 24;10(6):e0131320. doi: 10.1371/journal.pone.0131320 (PMC4479603; doi:10.1371/journal.pone.0131320)
Supplement: S3 Table — (DOCX) [file pone.0131320.s011.docx]

| Case | h [mm] | NS Von Mises Stress [%] | PPP Von Mises Stress [%] | PPH Von Mises Stress [%] | CV Von Mises Stress [%] | CPC Von Mises Stress [%] | SSP Von Mises Stress [%] | PF Von Mises Stress [%] | Max. displacement [%] |
| --- | --- | --- | --- | --- | --- | --- | --- | --- | --- |
| 1 | 2.5 | 0.08 | 13.27 | 6.07 | 0.31 | 43.97 | 18.22 | 70.95 | 1.47 |
| 2 | 5 | 0.12 | 6.41 | 3.90 | 0.44 | 34.32 | 12.39 | 61.25 | 0.57 |
| 3 | 7.5 | 1.03 | 1.95 | 4.47 | 0.51 | 21.27 | 7.93 | 46.94 | 0.02 |
| 4 | 10 | 1.03 | 4.16 | 3.18 | 0.88 | 14.07 | 5.92 | 29.26 | 0.26 |
| 5 | 12.5 | 0.77 | 3.98 | 3.63 | 1.62 | 6.24 | 2.72 | 16.27 | 0.37 |
| 6 | 15 | 0.44 | 2.70 | 2.14 | 0.29 | 2.67 | 0.81 | 4.67 | 0.32 |
| 7 | 17.5 | 0.00 | 0.00 | 0.00 | 0.00 | 0.00 | 0.00 | 0.00 | 0.00 |
| 8 | 20 | 1.14 | 0.32 | 1.53 | 2.33 | 1.05 | 0.62 | 2.84 | 0.28 |
| 9 | 22.5 | 0.26 | 1.09 | 1.76 | 0.41 | 3.53 | 0.72 | 2.58 | 0.89 |
| 10 | 25 | 0.28 | 2.39 | 0.84 | 3.13 | 4.46 | 1.32 | 0.76 | 1.39 |
| 11 | 27.5 | 1.73 | 0.68 | 1.00 | 4.17 | 5.04 | 1.80 | 0.40 | 2.02 |
| 12 | 30 | 1.23 | 0.54 | 1.53 | 3.95 | 8.50 | 2.12 | 1.78 | 2.52 |
| 13 | 32.5 | 2.54 | 0.30 | 1.64 | 3.41 | 6.59 | 2.06 | 6.23 | 2.96 |
| 14 | 35 | 1.14 | 0.05 | 0.68 | 12.20 | 7.90 | 2.25 | 6.34 | 3.38 |
| 15 | 37.5 | 0.79 | 2.93 | 1.06 | 6.11 | 11.05 | 2.30 | 5.67 | 3.46 |
| 16 | 40 | 1.18 | 5.18 | 1.09 | 10.14 | 11.63 | 2.18 | 5.94 | 3.51 |
| 17 | 42.5 | 1.17 | 0.90 | 1.15 | 10.22 | 12.06 | 2.23 | 5.54 | 3.31 |
| 18 | 45 | 0.41 | 4.06 | 2.17 | 12.08 | 14.81 | 2.22 | 9.37 | 2.93 |

**Table S3 Percent differences of Von Mises stress and displacements** obtained for the parameterization of the position of the orbits (h) under a bilateral bite in relationship its original position (h=17.5 mm)
