# Supplementary material for: 3D Computational Mechanics Elucidate the Evolutionary Implications of Orbit Position and Size Diversity of Early Amphibians
Source: PLoS One. 2015 Jun 24;10(6):e0131320. doi: 10.1371/journal.pone.0131320 (PMC4479603; doi:10.1371/journal.pone.0131320)
Supplement: S4 Table — (DOCX) [file pone.0131320.s012.docx]

| Case | S | NS Von Mises Stress [%] | PPP Von Mises Stress [%] | PPH Von Mises Stress [%] | CV Von Mises Stress [%] | PC Von Mises Stress [%] | SSP Von Mises Stress [%] | PF Von Mises Stress [%] | Max. displacement [%] |
| --- | --- | --- | --- | --- | --- | --- | --- | --- | --- |
| 1 | 0.125 | 3.00 | 2.26 | 2.51 | 6.77 | 9.44 | 2.14 | 9.34 | 5.26 |
| 2 | 0.25 | 2.55 | 3.24 | 1.61 | 6.87 | 8.76 | 2.10 | 3.88 | 5.06 |
| 3 | 0.375 | 1.91 | 2.36 | 3.28 | 9.42 | 7.58 | 1.77 | 7.17 | 4.52 |
| 4 | 0.5 | 1.25 | 4.72 | 2.56 | 4.69 | 7.20 | 1.19 | 5.47 | 4.09 |
| 5 | 0.625 | 1.46 | 1.48 | 1.20 | 5.85 | 5.65 | 1.17 | 4.24 | 3.34 |
| 6 | 0.75 | 0.26 | 0.04 | 0.80 | 4.51 | 3.35 | 1.03 | 1.42 | 2.37 |
| 7 | 0.875 | 0.86 | 0.58 | 0.23 | 2.64 | 2.82 | 0.49 | 0.25 | 1.34 |
| 8 | 1 | 0.00 | 0.00 | 0.00 | 0.00 | 0.00 | 0.00 | 0.00 | 0.00 |
| 9 | 1.125 | 1.97 | 0.52 | 2.64 | 9.82 | 1.97 | 0.57 | 1.58 | 1.57 |
| 10 | 1.25 | 2.16 | 0.27 | 3.96 | 2.98 | 8.61 | 1.04 | 1.96 | 3.20 |
| 11 | 1.375 | 3.51 | 1.02 | 5.48 | 0.48 | 10.67 | 1.91 | 10.58 | 5.21 |
| 12 | 1.5 | 4.89 | 3.41 | 7.12 | 6.64 | 15.49 | 2.74 | 22.71 | 7.71 |
| 13 | 1.625 | 7.37 | 4.67 | 6.08 | 4.32 | 27.68 | 3.66 | 30.60 | 10.50 |

**Table S4 Percent differences of Von Mises stress and displacements** obtained for the parameterization of the size of the orbits (S) under a bilateral bite in relationship with the original size of the orbits (S=1).
