# Supplementary material for: 3D Computational Mechanics Elucidate the Evolutionary Implications of Orbit Position and Size Diversity of Early Amphibians
Source: PLoS One. 2015 Jun 24;10(6):e0131320. doi: 10.1371/journal.pone.0131320 (PMC4479603; doi:10.1371/journal.pone.0131320)
Supplement: S5 Table — (DOCX) [file pone.0131320.s013.docx]

| Case | h [mm] | NS Von Mises Stress [MPa] | PPP Von Mises Stress [MPa] | PPH Von Mises Stress [MPa] | CV Von Mises Stress [MPa] | PC Von Mises Stress [MPa] | SSP Von Mises Stress [MPa] | PF Von Mises Stress [MPa] | Max. displacement [mm] |
| --- | --- | --- | --- | --- | --- | --- | --- | --- | --- |
| 1 | 2.5 | 0.0107 | 0.0167 | 0.0446 | 0.0074 | 0.0435 | 0.0380 | 0.0107 | 0.0073 |
| 2 | 5 | 0.0107 | 0.0163 | 0.0445 | 0.0091 | 0.0436 | 0.0339 | 0.0107 | 0.0073 |
| 3 | 7.5 | 0.0107 | 0.0157 | 0.0447 | 0.0078 | 0.0421 | 0.0320 | 0.0107 | 0.0074 |
| 4 | 10 | 0.0106 | 0.0157 | 0.0453 | 0.0075 | 0.0418 | 0.0232 | 0.0106 | 0.0073 |
| 5 | 12.5 | 0.0107 | 0.0156 | 0.0463 | 0.0076 | 0.0419 | 0.0164 | 0.0107 | 0.0072 |
| 6 | 15 | 0.0106 | 0.0150 | 0.0453 | 0.0079 | 0.0415 | 0.0129 | 0.0106 | 0.0073 |
| 7 | 17.5 | 0.0107 | 0.0150 | 0.0448 | 0.0077 | 0.0421 | 0.0127 | 0.0107 | 0.0073 |
| 8 | 20 | 0.0106 | 0.0153 | 0.0444 | 0.0072 | 0.0412 | 0.0127 | 0.0106 | 0.0072 |
| 9 | 22.5 | 0.0106 | 0.0152 | 0.0442 | 0.0072 | 0.0412 | 0.0126 | 0.0106 | 0.0072 |
| 10 | 25 | 0.0108 | 0.0147 | 0.0448 | 0.0075 | 0.0415 | 0.0125 | 0.0108 | 0.0073 |
| 11 | 27.5 | 0.0108 | 0.0148 | 0.0455 | 0.0076 | 0.0415 | 0.0126 | 0.0108 | 0.0072 |
| 12 | 30 | 0.0106 | 0.0154 | 0.0438 | 0.0077 | 0.0421 | 0.0129 | 0.0106 | 0.0072 |
| 13 | 32.5 | 0.0110 | 0.0146 | 0.0451 | 0.0080 | 0.0415 | 0.0127 | 0.0110 | 0.0072 |
| 14 | 35 | 0.0110 | 0.0149 | 0.0459 | 0.0077 | 0.0408 | 0.0126 | 0.0110 | 0.0071 |
| 15 | 37.5 | 0.0111 | 0.0147 | 0.0451 | 0.0078 | 0.0413 | 0.0127 | 0.0111 | 0.0072 |
| 16 | 40 | 0.0111 | 0.0152 | 0.0443 | 0.0073 | 0.0410 | 0.0127 | 0.0111 | 0.0071 |
| 17 | 42.5 | 0.0111 | 0.0152 | 0.0452 | 0.0075 | 0.0407 | 0.0128 | 0.0111 | 0.0072 |
| 18 | 45 | 0.0113 | 0.0153 | 0.0449 | 0.0068 | 0.0408 | 0.0128 | 0.0113 | 0.0072 |

**Table S5 Von Mises stress and displacements** obtained for the parameterization of the position of the orbit (h) during the skull-raising loading
