# Supplementary material for: 3D Computational Mechanics Elucidate the Evolutionary Implications of Orbit Position and Size Diversity of Early Amphibians
Source: PLoS One. 2015 Jun 24;10(6):e0131320. doi: 10.1371/journal.pone.0131320 (PMC4479603; doi:10.1371/journal.pone.0131320)
Supplement: S6 Table — (DOCX) [file pone.0131320.s014.docx]

| Case | S | NS Von Mises Stress [MPa] | PPP Von Mises Stress [MPa] | PPH Von Mises Stress [MPa] | CV Von Mises Stress [MPa] | PC Von Mises Stress [MPa] | SSP Von Mises Stress [MPa] | PF Von Mises Stress [MPa] | Max. displacement [mm] |
| --- | --- | --- | --- | --- | --- | --- | --- | --- | --- |
| 1 | 0.125 | 0.0105 | 0.0149 | 0.0454 | 0.0082 | 0.0396 | 0.0137 | 0.0266 | 0.0074 |
| 2 | 0.25 | 0.0104 | 0.0148 | 0.0455 | 0.0086 | 0.0395 | 0.0137 | 0.0268 | 0.0074 |
| 3 | 0.375 | 0.0105 | 0.0149 | 0.0454 | 0.0085 | 0.0410 | 0.0135 | 0.0274 | 0.0074 |
| 4 | 0.5 | 0.0105 | 0.0150 | 0.0452 | 0.0086 | 0.0403 | 0.0134 | 0.0289 | 0.0074 |
| 5 | 0.625 | 0.0105 | 0.0149 | 0.0453 | 0.0083 | 0.0409 | 0.0132 | 0.0287 | 0.0073 |
| 6 | 0.75 | 0.0108 | 0.0149 | 0.0464 | 0.0089 | 0.0395 | 0.0130 | 0.0212 | 0.0073 |
| 7 | 0.875 | 0.0108 | 0.0150 | 0.0443 | 0.0084 | 0.0403 | 0.0128 | 0.0278 | 0.0073 |
| 8 | 1 | 0.0109 | 0.0150 | 0.0442 | 0.0085 | 0.0406 | 0.0128 | 0.0267 | 0.0073 |
| 9 | 1.125 | 0.0109 | 0.0150 | 0.0437 | 0.0084 | 0.0401 | 0.0126 | 0.0285 | 0.0073 |
| 10 | 1.25 | 0.0112 | 0.0152 | 0.0433 | 0.0085 | 0.0393 | 0.0129 | 0.0275 | 0.0072 |
| 11 | 1.375 | 0.0113 | 0.0153 | 0.0431 | 0.0081 | 0.0403 | 0.0131 | 0.0266 | 0.0072 |
| 12 | 1.5 | 0.0116 | 0.0156 | 0.0422 | 0.0087 | 0.0403 | 0.0135 | 0.0282 | 0.0072 |
| 13 | 1.625 | 0.0119 | 0.0159 | 0.0415 | 0.0091 | 0.0400 | 0.0142 | 0.0250 | 0.0072 |

**Table S6 Von Mises stress and displacements** obtained for the parameterization of the size of the orbit (S) during the skull-raising loading
