# Supplementary material for: 3D Computational Mechanics Elucidate the Evolutionary Implications of Orbit Position and Size Diversity of Early Amphibians
Source: PLoS One. 2015 Jun 24;10(6):e0131320. doi: 10.1371/journal.pone.0131320 (PMC4479603; doi:10.1371/journal.pone.0131320)
Supplement: S7 Table — (DOCX) [file pone.0131320.s015.docx]

| Case | h [mm] | NS Von Mises Stress [%] | PPP Von Mises Stress [%] | PPH Von Mises Stress [%] | CV Von Mises Stress [%] | PC Von Mises Stress [%] | SSP Von Mises Stress [%] | PF Von Mises Stress [%] | Max. displacement [%] |
| --- | --- | --- | --- | --- | --- | --- | --- | --- | --- |
| 1 | 2.5 | 0.23 | 10.42 | 0.58 | 4.61 | 3.22 | 66.59 | 29.96 | 0.21 |
| 2 | 5 | 0.01 | 8.13 | 0.74 | 14.84 | 3.47 | 62.51 | 28.79 | 0.33 |
| 3 | 7.5 | 0.02 | 4.55 | 0.28 | 1.56 | 0.09 | 60.25 | 28.49 | 0.66 |
| 4 | 10 | 0.72 | 4.44 | 0.97 | 3.42 | 0.58 | 45.32 | 4.42 | 0.27 |
| 5 | 12.5 | 0.11 | 3.66 | 3.19 | 2.07 | 0.34 | 22.57 | 6.39 | 1.06 |
| 6 | 15 | 0.96 | 0.20 | 1.07 | 2.46 | 1.31 | 1.71 | 3.70 | 0.10 |
| 7 | 17.5 | 0.00 | 0.00 | 0.00 | 0.00 | 0.00 | 0.00 | 0.00 | 0.00 |
| 8 | 20 | 1.35 | 1.68 | 0.90 | 6.68 | 2.19 | 0.36 | 1.86 | 1.35 |
| 9 | 22.5 | 0.51 | 1.20 | 1.35 | 6.57 | 1.97 | 0.86 | 3.09 | 1.44 |
| 10 | 25 | 0.71 | 1.80 | 0.00 | 2.55 | 1.39 | 2.03 | 5.10 | 0.75 |
| 11 | 27.5 | 1.26 | 1.67 | 1.46 | 1.71 | 1.25 | 0.62 | 9.60 | 0.79 |
| 12 | 30 | 1.03 | 2.57 | 2.39 | 0.67 | 0.15 | 1.30 | 10.58 | 1.21 |
| 13 | 32.5 | 3.01 | 2.43 | 0.61 | 3.95 | 1.23 | 0.45 | 6.06 | 1.22 |
| 14 | 35 | 2.38 | 0.50 | 2.25 | 0.24 | 3.19 | 0.72 | 2.92 | 2.25 |
| 15 | 37.5 | 3.68 | 1.79 | 0.54 | 0.80 | 1.79 | 0.09 | 6.97 | 1.26 |
| 16 | 40 | 3.16 | 1.56 | 1.28 | 5.14 | 2.70 | 0.20 | 8.73 | 2.24 |
| 17 | 42.5 | 3.84 | 1.22 | 0.89 | 3.49 | 3.33 | 0.55 | 23.67 | 2.10 |
| 18 | 45 | 5.07 | 2.07 | 0.11 | 13.80 | 3.04 | 0.86 | 2.82 | 1.93 |

**Table S7 Percent differences of Von Mises Stress and displacements** obtained for the parameterization of the position of the orbits (h) during the skull-raising loading in relationship with its original position (h=17.5 mm)

.
