# Supplementary material for: 3D Computational Mechanics Elucidate the Evolutionary Implications of Orbit Position and Size Diversity of Early Amphibians
Source: PLoS One. 2015 Jun 24;10(6):e0131320. doi: 10.1371/journal.pone.0131320 (PMC4479603; doi:10.1371/journal.pone.0131320)
Supplement: S8 Table — (DOCX) [file pone.0131320.s016.docx]

| Case | S | NS Von Mises Stress [%] | PPP Von Mises Stress [%] | PPH Von Mises Stress [%] | CV Von Mises Stress [%] | PC Von Mises Stress [%] | SSP Von Mises Stress [%] | PF Von Mises Stress [%] | Max. displacement [%] |
| --- | --- | --- | --- | --- | --- | --- | --- | --- | --- |
| 1 | 0.125 | 3.63 | 0.89 | 2.62 | 3.53 | 2.55 | 6.61 | 0.40 | 1.17 |
| 2 | 0.25 | 4.60 | 1.04 | 2.75 | 1.12 | 2.69 | 6.26 | 0.28 | 1.33 |
| 3 | 0.375 | 3.54 | 0.60 | 2.53 | 0.17 | 0.98 | 5.34 | 2.49 | 1.06 |
| 4 | 0.5 | 3.25 | 0.29 | 2.19 | 1.04 | 0.68 | 4.11 | 7.40 | 1.05 |
| 5 | 0.625 | 3.29 | 0.53 | 2.44 | 3.14 | 0.69 | 2.78 | 6.76 | 0.68 |
| 6 | 0.75 | 1.07 | 0.75 | 4.61 | 3.84 | 2.66 | 1.30 | 25.94 | 0.60 |
| 7 | 0.875 | 0.53 | 0.22 | 0.16 | 0.79 | 0.77 | 0.34 | 3.93 | 0.17 |
| 8 | 1 | 0.00 | 0.00 | 0.00 | 0.00 | 0.00 | 0.00 | 0.00 | 0.00 |
| 9 | 1.125 | 0.23 | 0.22 | 1.17 | 0.76 | 1.21 | 1.44 | 6.12 | 0.32 |
| 10 | 1.25 | 3.01 | 1.18 | 2.07 | 0.32 | 3.38 | 0.29 | 2.97 | 0.61 |
| 11 | 1.375 | 3.93 | 2.21 | 2.66 | 5.41 | 0.86 | 2.28 | 0.62 | 1.28 |
| 12 | 1.5 | 6.19 | 3.71 | 4.90 | 2.47 | 0.78 | 4.89 | 5.25 | 0.98 |
| 13 | 1.625 | 8.56 | 5.59 | 6.70 | 6.85 | 1.62 | 9.78 | 6.83 | 1.50 |

**Table S8 Percent differences of Von Mises Stress and displacements** obtained for the parameterization of the size of the orbit (S) during the skull-raising loading in relationship with the original size of the orbits (S=1).

.
