# Supplementary material for: 3D Computational Mechanics Elucidate the Evolutionary Implications of Orbit Position and Size Diversity of Early Amphibians
Source: PLoS One. 2015 Jun 24;10(6):e0131320. doi: 10.1371/journal.pone.0131320 (PMC4479603; doi:10.1371/journal.pone.0131320)
Supplement: S9 Table — (DOCX) [file pone.0131320.s017.docx]

|  | NS Von Mises Stress [MPa] | PPP Von Mises Stress [MPa] | PPH Von Mises Stress [MPa] | CV Von Mises Stress [MPa] | CP Von Mises Stress [MPa] | SSP Von Mises Stress [MPa] | PF Von Mises Stress [MPa] | Max. displacement [mm] |
| --- | --- | --- | --- | --- | --- | --- | --- | --- |
| S, Bilateral Bite | | | | | | | | |
| Breusch-Pagan (p) | 0,1897 | 0,9841 | 0,6422 | 0,8409 | 0,2634 | 0,2783 | 0,0308 | 0,3297 |
| Slope a: | -0,2056 | 0,1692 | 0,1373 | -0,3238 | -0,0761 | 0,0862 | 1,3404 | 0,0067 |
| Std. error a: | 0,0190 | 0,0271 | 0,0152 | 0,0823 | 0,0070 | 0,0070 | 0,2852 | 0,0007 |
| Intercept b: | 3,6310 | 3,4695 | 1,8259 | 4,1850 | 0,4534 | 2,2150 | 4,2318 | 0,0584 |
| Std. error b: | 0,0189 | 0,0269 | 0,0151 | 0,0816 | 0,0069 | 0,0070 | 0,2829 | 0,0007 |
| p (uncorr.): | 3.4375E-07 | 6.3625E-05 | 2.0551E-06 | 0,0023 | 3.1323E-07 | 9.1262E-08 | 0,0006 | 7.0139E-07 |
| r2 | 0,9137 | 0,7796 | 0,8809 | 0,5848 | 0,9151 | 0,9321 | 0,6676 | 0,9018 |
| h, Bilateral bite | | | | | | | | |
| Breusch-Pagan (p) | 0,9978 | 0,0962 | 0,6676 | 0,4011 | 0,0140 | 0,0633 | 0,0400 | 0,0413 |
| Slope a: | -0,0010 | -0,0100 | -0,0034 | 0,0130 | 0,0038 | -0,0092 | 0,0345 | 6.2704E-05 |
| Intercept b: | 3,4777 | 3,8914 | 2,0194 | 3,7198 | 0,3015 | 2,5564 | 3,7976 | 0,0636 |
| Std. error a: | 0,0005 | 0,0019 | 0,0004 | 0,0018 | 0,0003 | 0,0018 | 0,0102 | 9.8901E-06 |
| Std. error b: | 0,0124 | 0,0514 | 0,0122 | 0,0495 | 0,0092 | 0,0486 | 0,2759 | 0,0003 |
| p (uncorr.): | 0,0383 | 7.499E-05 | 1.1574E-06 | 2.3901E-06 | 7.0124E-09 | 0,0001 | 0,0038 | 9.8173E-06 |
| r2 | 0,2417 | 0,6352 | 0,7810 | 0,7606 | 0,8836 | 0,6215 | 0,4175 | 0,7153 |
| S, Skull-raising | | | | | | | | |
| Breusch-Pagan (p) | 0,5153 | 0,0859 | 0,7141 | 0,1349 | 0,1140 | 0,0770 | 0,8849 | 1,0000 |
| Slope a: | 0,0009 | 0,0005 | -0,0026 | 0,0002 | 4.3956E-06 | -0,0001 | -0,0002 | -0,0002 |
| Intercept b: | 0,0101 | 0,0146 | 0,0466 | 0,0084 | 0,0401 | 0,0134 | 0,0271 | 0,0074 |
| Std. error a: | 0,0001 | 0,0001 | 0,0004 | 0,0002 | 0,0003 | 0,0003 | 0,0013 | 1.6764E-05 |
| Std. error b: | 9.9969E-05 | 0,0001 | 0,0004 | 0,0002 | 0,0003 | 0,0003 | 0,0012 | 1.6633E-05 |
| p (uncorr.): | 2.1267E-06 | 0,0004 | 4.5471E-05 | 0,0898 | 1.5845E-05 | 0,0138 | 0,0027 | 1.3119E-06 |
| r2 | 0,8801 | 0,6984 | 0,7924 | 0,3198 | 0,9897 | 0,7024 | 0,8666 | 0,8901 |
| h, Skull-raising | | | | | | | | |
| Breusch-Pagan (p) | 0,8305 | 0,5274 | 0,8778 | 0,1611 | 0,0110 | 0,0054 | 0,0076 | 1 |
| Slope a: | 1.3498E-05 | 0,0000 | 8.6687E-07 | -1.4159E-05 | -4.8669E-05 | -0,0005 | -0,0003 | -4.2105E-06 |
| Intercept b: | 0,0105 | 0,0160 | 0,0449 | 0,0080 | 0,0428 | 0,0284 | 0,0343 | 0,0073333 |
| Std. error a: | 2.4602E-06 | 0,0271 | 1.1506E-05 | 8.0106E-06 | 9.2255E-06 | 0,0001 | 6.0268E-05 | 9.7773E-07 |
| Std. error b: | 6.6576E-05 | 0,0269 | 0,0003 | 0,0002 | 0,0002 | 0,0029 | 0,0016 | 2.6458E-05 |
| p (uncorr.): | 4.9733E-05 | 0,0026 | 0,0004 | 0,1634 | 7.5417E-05 | 0,0004 | 0,0004 | 0,00054332 |
| r2 | 0,6530 | 0,4433 | 0,9409 | 0,0962 | 0,6350 | 0,5514 | 0,5534 | 0,53684 |

**Table S9 Statistics of the OLS regression** between orbit size and location and the stress variables and displacement

.
