# Supplementary material for: 3D Computational Mechanics Elucidate the Evolutionary Implications of Orbit Position and Size Diversity of Early Amphibians
Source: PLoS One. 2015 Jun 24;10(6):e0131320. doi: 10.1371/journal.pone.0131320 (PMC4479603; doi:10.1371/journal.pone.0131320)
Supplement: S10 Table — (DOCX) [file pone.0131320.s018.docx]

| PC | % explained variance | | | | |
| --- | --- | --- | --- | --- | --- |
|  | S, Bilateral bite | h, Bilateral bite | S, skull raising | S, skull raising without PF | h, skull-raising |
| 1 | 96,74 | 91,76 | 61,06 | 79,78 | 95,32 |
| 2 | 2,59 | 6,69 | 32,18 | 10,34 | 3,88 |
| 3 | 0,49 | 1,20 | 3,19 | 7,07 | 0,36 |
| 4 | 0,14 | 0,18 | 2,92 | 2,20 | 0,25 |
| 5 | 0,04 | 0,11 | 0,55 | 0,56 | 0,12 |
| 6 | 0,00 | 0,06 | 0,09 | 0,04 | 0,04 |
| 7 | 0,00 | 0,01 | 0,01 | - | 0,03 |

**Table S10 Percent of explained variable** for each PC at the different PCAs developed in this work.
