# Supplementary material for: 3D Computational Mechanics Elucidate the Evolutionary Implications of Orbit Position and Size Diversity of Early Amphibians
Source: PLoS One. 2015 Jun 24;10(6):e0131320. doi: 10.1371/journal.pone.0131320 (PMC4479603; doi:10.1371/journal.pone.0131320)
Supplement: S11 Table — (DOCX) [file pone.0131320.s019.docx]

| S, Bilateral bite | | | | | | | |
| --- | --- | --- | --- | --- | --- | --- | --- |
|  | PC 1 | PC 2 | PC 3 | PC 4 | PC 5 | PC 6 | PC 7 |
| NS | -0,118 | 0,045 | -0,586 | 0,463 | 0,477 | 0,424 | -0,137 |
| PPP | 0,102 | -0,011 | 0,508 | 0,843 | -0,135 | -0,026 | 0,038 |
| PPH | 0,074 | -0,102 | 0,517 | -0,188 | 0,825 | 0,013 | -0,033 |
| CV | -0,192 | 0,969 | 0,140 | -0,040 | 0,038 | 0,033 | -0,017 |
| CP | -0,044 | 0,043 | -0,194 | 0,103 | 0,196 | -0,328 | 0,895 |
| SSP | 0,049 | -0,044 | 0,222 | -0,163 | -0,182 | 0,843 | 0,420 |
| PF | 0,964 | 0,211 | -0,157 | -0,013 | 0,035 | 0,003 | -0,002 |
| h, Bilateral bite | | | | | | | |
| NS | -0,011 | -0,048 | 0,039 | 0,302 | 0,826 | -0,460 | -0,105 |
| PPP | -0,185 | -0,311 | 0,822 | -0,426 | 0,040 | -0,094 | -0,027 |
| PPH | -0,059 | -0,090 | 0,037 | 0,008 | 0,479 | 0,803 | 0,336 |
| CV | 0,100 | 0,902 | 0,400 | 0,060 | 0,051 | 0,084 | -0,062 |
| CP | 0,063 | 0,114 | -0,032 | -0,154 | -0,015 | -0,353 | 0,913 |
| SSP | -0,197 | -0,162 | 0,349 | 0,831 | -0,289 | 0,034 | 0,194 |
| PF | 0,954 | -0,202 | 0,195 | 0,097 | -0,017 | 0,047 | 0,001 |
| S, Skull raising | | | | | | | |
| NS | -0,003 | -0,308 | 0,047 | -0,062 | 0,091 | 0,685 | 0,650 |
| PPP | 0,013 | -0,202 | 0,070 | 0,145 | 0,095 | 0,610 | -0,743 |
| PPH | -0,244 | 0,896 | 0,008 | 0,097 | 0,097 | 0,338 | 0,062 |
| CV | -0,060 | -0,077 | 0,038 | 0,207 | 0,955 | -0,181 | 0,038 |
| CP | 0,118 | 0,054 | 0,987 | -0,059 | -0,027 | -0,060 | 0,017 |
| SSP | -0,022 | -0,075 | 0,052 | 0,957 | -0,232 | -0,052 | 0,140 |
| PF | 0,960 | 0,217 | -0,117 | 0,065 | 0,081 | 0,075 | 0,032 |
| S, Skull raising without PF | | | | | | | |
| NS | -0,285 | -0,118 | -0,014 | 0,454 | 0,715 | -0,433 | - |
| PPP | -0,194 | -0,071 | 0,171 | 0,205 | 0,320 | 0,885 | - |
| PPH | 0,936 | -0,079 | 0,100 | 0,199 | 0,260 | 0,039 | - |
| CV | -0,046 | -0,212 | 0,226 | 0,764 | -0,562 | -0,044 | - |
| CP | -0,001 | 0,918 | 0,357 | 0,166 | 0,026 | -0,044 | - |
| SSP | -0,060 | -0,296 | 0,884 | -0,317 | 0,053 | -0,153 | - |
| h, Skull raising | | | | | | | |
| NS | -0,008 | -0,033 | 0,007 | -0,129 | -0,250 | 0,216 | 0,934 |
| PPP | 0,051 | -0,015 | -0,150 | -0,066 | 0,544 | 0,820 | -0,052 |
| PPH | -0,012 | -0,109 | 0,923 | -0,280 | 0,238 | -0,011 | 0,017 |
| CV | 0,021 | 0,037 | 0,337 | 0,630 | -0,517 | 0,444 | -0,155 |
| CP | 0,072 | 0,073 | 0,067 | 0,698 | 0,562 | -0,287 | 0,315 |
| SSP | 0,879 | -0,472 | -0,033 | -0,005 | -0,052 | -0,036 | -0,015 |
| PF | 0,468 | 0,870 | 0,075 | -0,129 | -0,024 | 0,007 | 0,009 |

**Table S11 Loadings of the original variables** for each PC at the different PCAs developed in this work.
